# AP2 Regulates Thickveins Trafficking to Attenuate NMJ Growth Signaling in *Drosophila*

**DOI:** 10.1523/ENEURO.0044-22.2022

**Published:** 2022-10-11

**Authors:** Saumitra Dey Choudhury, Manish Kumar Dwivedi, Srikanth Pippadpally, Abhinandan Patnaik, Shirish Mishra, Raghu Padinjat, Vimlesh Kumar

**Affiliations:** 1 Department of Biological Sciences, Indian Institute of Science Education and Research (IISER) Bhopal, Bhopal, Madhya Pradesh 462 066, India; 2 National Centre for Biological Sciences, Bangalore, Karnataka 560065, India

**Keywords:** σ_2_-adaptin, BMP receptors, Rab11, synaptic growth, Thickveins

## Abstract

Compromised endocytosis in neurons leads to synapse overgrowth and altered organization of synaptic proteins. However, the molecular players and the signaling pathways which regulate the process remain poorly understood. Here, we show that σ2-adaptin, one of the subunits of the AP2-complex, genetically interacts with Mad, Medea and Dad (components of BMP signaling) to control neuromuscular junction (NMJ) growth in *Drosophila*. Ultrastructural analysis of *σ2-adaptin* mutants show an accumulation of large vesicles and membranous structures akin to endosomes at the synapse. We found that mutations in *σ2-adaptin* lead to an accumulation of Tkv receptors at the presynaptic membrane. Interestingly, the level of small GTPase Rab11 was significantly reduced in the *σ2-adaptin* mutant synapses. However, expression of Rab11 does not restore the synaptic defects of *σ2-adaptin* mutations. We propose a model in which AP2 regulates Tkv internalization and endosomal recycling to control synaptic growth.

## Significance Statement

Understanding the regulation of synaptic development and refinement could provide mechanistic insights into the neural basis of fundamental processes such as learning, memory and adaptation. In this study, we describe the role of σ_2_-adaptin in attenuating neuromuscular junction (NMJ) growth signaling and show its involvement in the trafficking of Thickveins receptor at the *Drosophila* NMJ. σ_2_-Adaptin interacts genetically with the BMP pathway components and NMJ synapses lacking σ_2_-adaptin show reduced levels of the endosomal recycling marker, Rab11. Our study contributes to a broader understanding of AP2 complex-dependent regulation of synaptic growth signaling, which might have implications in neurodevelopment under conditions where endocytosis and trafficking of the BMP receptors are perturbed.

## Introduction

Synapse development and refinement is an interplay of multiple signaling pathways, namely, the Bone morphogenetic protein (BMP), Wingless (Wnt), and Ubiquitin-Proteasome-mediated protein degradation (Highwire-Wallenda) pathways (McCabe et al., 2003, 2004; Franco et al., 2004; Collins et al., 2006; Ball et al., 2010; Budnik and Salinas, 2011; Piccioli and Littleton, 2014; Choudhury et al., 2016). Besides, there are also pathways mediated by endocytic, cytoskeletal, and actin regulatory proteins (Franco et al., 2004; Piccioli and Littleton, 2014; Choudhury et al., 2016). Understanding the crosstalk among them is crucial to our understanding of the processes that regulate synapse development, refinement, and plasticity (Deshpande and Rodal, 2016). BMP signaling pathway at the *Drosophila* neuromuscular junction (NMJ) synapses is a well-studied growth-promoting pathway (Marqués et al., 2002; McCabe et al., 2003; O’Connor-Giles et al., 2008; Ball et al., 2010). The canonical BMP signaling is dependent on phosphorylated Smad (pMad in *Drosophila*) and its translocation into the motor neuron nuclei, followed by the transcription of BMP target genes (Keshishian and Kim, 2004; O’Connor-Giles et al., 2008; Ball et al., 2010; Kim and Marqués, 2012; Sulkowski et al., 2016; Vuilleumier et al., 2019).

At the *Drosophila* NMJ, the retrograde BMP signaling is initiated by secretion of Glass bottom boat (Gbb) from the muscle and the motor neurons (Goold and Davis, 2007). Gbb binds to Wit (a Type II receptor) as well as Tkv and Sax (Type I receptors) at the presynaptic nerve terminals to control NMJ growth and function (Aberle et al., 2002; Marqués et al., 2002; McCabe et al., 2003). Gbb binding to Wit triggers the tetramerization of BMP receptors that, in turn, phosphorylates the Smad transcription factor, Mothers against decapentaplegic (Mad). Following BMP activation, these receptors are endocytosed for retrograde transport to the motor neuron nuclei in the soma (Rodal et al., 2011; Smith et al., 2012; Vanlandingham et al., 2013).

Multiple studies have shown a tight correlation between defective endocytosis, altered synapse growth, and elevated synaptic pMad levels, implicating increased BMP signaling in synaptic development (O’Connor-Giles et al., 2008; Ball et al., 2010; Nahm et al., 2010; Shi et al., 2013; Piccioli and Littleton, 2014). One such study has shown that Nwk, an F-BAR and SH3 domain-containing protein that negatively regulates synaptic growth, interacts with Tkv along with Dap160 and Dynamin (both endocytic proteins) to attenuate retrograde BMP signaling during NMJ growth (O’Connor-Giles et al., 2008). Endocytic and endosomal pathways are, therefore, critical to controlling both the activity and localization of signaling proteins that regulate synaptic growth (Rodal et al., 2011; Cosker and Segal, 2014). Defects in intracellular trafficking can also lead to enhanced signaling from the cellular compartments (like endosomes), affecting the synapse development (Di Fiore and De Camilli, 2001; Dubois et al., 2001; Sweeney and Davis, 2002; Rodal et al., 2011). In the neuronal context, the efficacy of intercellular signaling is regulated by the trafficking of activated receptor/ligand complexes following endocytosis from the presynaptic membrane.

Tightly regulated endocytic transport of BMP receptors relies on the spatiotemporal regulation of Rab GTPase function (Kelly et al., 2012). The Rab-family of GTPases regulates the progression of receptor endocytosis and participates in the successive steps of membrane maturation, receptor transport, and turnover (Horgan and McCaffrey, 2011). In particular, Rab5 regulates vesicle formation and is associated with early endosomes, while Rab7 and Rab11 associate with late and recycling endosomes, respectively (Chavrier et al., 1990; Ullrich et al., 1996). Endosomal trafficking of BMP signaling complexes at the nerve terminals is known to fine-tune the intensity and persistence of BMP signaling (Cosker and Segal, 2014; Deshpande and Rodal, 2016). Altered distribution or misregulation of Rab11 has been shown to suppress Tkv trafficking from early endosome to presynaptic membrane resulting in elevated BMP signaling (O’Connor-Giles et al., 2008; Rodal et al., 2008; Liu et al., 2014; Deshpande and Rodal, 2016).

An important, yet enigmatic question, is the correlation between defective Clathrin-mediated endocytosis (CME) and aberrant synaptic growth. For instance, it is not known whether specific modes of endocytosis externalize specific cargos whose trafficking defect perturbs synaptic signaling. CME, the major endocytic pathway, is required not only for basal synaptic transmission at nerve terminals but also for synapse development (Koh et al., 2004; Dickman et al., 2006; Choudhury et al., 2016). For instance, perturbations in CME resulting from mutations in Dynamin, AP2 subunits, Endo, or Synj all exhibit NMJ structural defects resulting in increased number but decreased size of synaptic boutons in *Drosophila* (Dickman et al., 2006; Choudhury et al., 2016). It is unclear whether the NMJ structural defects associated with the endocytic mutants is a consequence of deficient endosomal trafficking leading to aberrant synaptic signaling. It is likely that perturbing CME deregulates signaling modules of BMP pathway that leads to elevated pMad in the endocytic mutants (O’Connor-Giles et al., 2008; Choudhury et al., 2016).

In central synapses, AP2-dependent CME is dispensable for membrane regeneration from the presynaptic plasma membrane following high-frequency nerve stimulation (Kononenko et al., 2014). However, the critical role of CME in generating vesicles from endosome-like structures following bulk membrane endocytosis cannot be ruled out (Watanabe et al., 2014). Previous studies support a model in which compromised CME can lead to defective signalosome trafficking by trapping signaling molecules in endosomes or intermediate structures of the endosomal pathway (Dubois et al., 2001; Lloyd et al., 2002; Sweeney and Davis, 2002; Wang et al., 2007; Papagiannouli et al., 2019; Joseph et al., 2020). Another study has shown clathrin-independent role of the AP2 in the endocytic retrieval of select synaptic vesicle (SV) cargos from the presynaptic cell surface (López-Hernández et al., 2021).

Our previous study has shown elevated levels of synaptic as well as motor-nuclei pMad in *σ2-adaptin* mutants (Choudhury et al., 2016). In order to investigate the underlying signaling mechanisms leading to elevated pMad levels, we performed epistatic interactions between *σ2-adaptin* mutants with the components of BMP signaling. Introducing a mutant copy of *tkv* in *σ2-adaptin* mutants significantly reduces the NMJ overgrowth. Conversely, introducing a mutant copy of inhibitory Smad, *Dad* in a heterozygous *σ2-adaptin* background leads to NMJ overgrowth. Ultrastructural analysis of NMJ revealed accumulation of large vesicles and supports a role of σ2-adaptin in generating signalosomes containing vesicles, possibly from endosomal structures. Further analysis of vesicular trafficking using endosomal markers shows that Rab11 is reduced in *σ2-adaptin* mutant NMJ synapses. Thus, our studies reveal a novel function of σ2-adaptin in attenuating BMP signaling by facilitating trafficking and recycling of the Tkv receptor.

## Materials and Methods

### Fly stock

Flies were grown and maintained at 25°C temperature in a standard cornmeal medium as described earlier (Choudhury et al., 2016). Both males and females were used for experiments in the current study. *w^1118^* was used as control unless otherwise stated. Two σ2-adaptin alleles were used: *AP2σ^ang7^,*which is a hypomorphic allele obtained through a *P*-element mobilization of *Synd^EP877^* located 2416 bp upstream of the Syndapin ORF, and *AP2σ^KG02457^,* which is a *P*-element insertion in the third exon of the σ2-adaptin ORF making it a null allele (Choudhury et al., 2016). All the mutants, controls, and rescued larvae of either sex were grown under noncrowded conditions on apple agar plates with yeast paste dollop. The following stocks were obtained from Bloomington *Drosophila* Stock Center (BDSC): *tkv^7^* (BL3242), *Med^13^* (BL7340), *wit^A12^* (BL5173), *Dad^j1E4^* (BL10305), *AP2σ^KG02457^* (BL13478), *UAS-Rab11-GFP* (BL8506), *Mad^k00237^* (BL10474), *α-adaptin* dsRNA (BL32866), *Clc* dsRNA (BL27496), *Synj^1^* (BL24883), *Synj^2^* (BL24884). Other lines used in this study are: *D42-Gal4* (Yeh et al., 1995; Sanyal, 2009), *UAS-tkv-EGFP* (BL51653; Ayyaz et al., 2015), *UAS-YFP-Rab11^S25N^* (BL9792; Guan et al., 2020), *UAS-Rab11^Q70L^-GFP* (BL23260; Sorvina et al., 2016), *Rab11^ex2^* and *Rab11^93Bi^* (Khodosh et al., 2006).

### Antibodies and immunocytochemistry

Wandering third instar larvae were dissected in cold calcium-free HL3 saline (70 mm NaCl, 5 mm KCl, 20 mm MgCl_2_, 10 mm NaHCO_3_, 5 mm Trehalose, 115 mm sucrose, and 5 mm HEPES, pH 7.2) to expose the NMJs and fixed in 4% paraformaldehyde in PBS (pH 7.2) for 30 min at room temperature. Fillets were then washed in PBS containing 0.15% Triton X-100, blocked for 1 h with 5% bovine serum albumin (BSA), followed by overnight incubation with primary antibody at 4°C. The monoclonal antibody anti-CSP (1:100) was obtained from the Developmental Studies Hybridoma Bank (DSHB). The polyclonal antibody against Rab5 (1:500; Mottola et al., 2010) was a gift from Marino Zerial, Max Planck Institute, Germany. The polyclonal Rab7 (1:500) and Rab11 (1:500; Tanaka and Nakamura, 2008; West et al., 2015) antibodies were a gift from Tsubasa Tanaka, RIKEN Center for Developmental Biology, Japan. The pMad antibody was a gift from Peter ten Dijke and used at 1:1000 dilution (Persson et al., 1998). The secondary antibodies conjugated to Alexa Fluor 488 and Alexa Fluor 568 (Invitrogen, Thermo Fisher Scientific) were used at 1:800 dilution. The Alexa Fluor 488 or Rhodamine-conjugated anti-HRP (Jackson ImmunoResearch) were used at 1:800 dilution. Stained larval fillets were mounted in VECTASHIELD (Vector Laboratories). All the images were captured with a laser scanning confocal microscope (LSM780, Carl Zeiss or FV3000, Olympus Corporation).

### Electrophysiology

All intracellular recordings were performed on wandering third instar larvae as described previously (Choudhury et al., 2016). Briefly, HL3 buffer containing 1.5 mm Ca^2+^ was used for larval dissection. Recordings from muscle 6 of A2 hemisegment were performed using sharp glass electrodes having a resistance of 20–25 MΩ. Miniature excitatory junction potentials (mEJPs) were recorded for 60 s, followed by recordings of EJPs at 1 Hz stimulation. For High-frequency recording, nerves were stimulated at 10 Hz, and EJPs were recorded for 5 min. For recording EJPs, stimulation pulse was delivered using Grass S88 stimulator (Grass Instruments, Astro-Med, Inc). The signals were amplified using Axoclamp 900A, digitized using Digidata 1440A, and acquired using pClamp10 software (Molecular Devices). Muscles with resting membrane potential between −60 and −75 mV were used for analysis. The data were analyzed using the Mini Analysis program (Synaptosoft, Decatur).

### Intensity profile

Confocal images of muscle four NMJ at A2 hemisegment were used to plot the intensity profile. A single bouton section was used, and the intensities of Tkv and HRP were analyzed by drawing a line across the bouton using Fiji/ImageJ software. The graph was plotted in Microsoft Excel using the intensity values obtained from Fiji/ImageJ software. As the intensity of Tkv in control was too low to plot the graph, all the intensity values were multiplied two-fold.

### Electron microscopy

TEM was performed as described previously (Deivasigamani et al., 2015). Briefly, third instar larvae were dissected in cold PBS. The larval fillets were then fixed in 0.12 m cacodylate buffer containing 2% glutaraldehyde for 10 min at room temperature, transferred to a fresh fixative, and kept overnight at 4°C. The fillets were postfixed for 1 h with 2% osmium tetroxide (OsO_4_) solution prepared in 0.12 m cacodylate buffer. The samples were rinsed with 0.12 m cacodylate buffer followed by washes with distilled water to avoid precipitation of cacodylate with Uranyl acetate. Subsequently, the samples were subjected to en bloc staining with 2% uranyl acetate. The stained fillets were again washed with distilled water and dehydrated using graded solutions of ethanol before final infiltration of the samples through propylene oxide for 30 min. Stained and dehydrated fillets were embedded in epoxy resin and hardened overnight at 60°C. Muscles embedded in epoxy resin were sectioned at 60 nm. Ultrathin sections of the muscles stained with 2% uranyl acetate (in 70% ethanol) and 1% aqueous lead citrate were examined at 120 KV on a Tecnai G2 Spirit BioTWIN (FEI) electron microscope. The number of synaptic vesicles per bouton were counted manually using the Multi-point tool in ImageJ/Fiji software and then divided by their respective bouton areas to obtain the vesicle density/μm^2^ area of a bouton. For vesicle size, diameters of at least 100 vesicles from 10 bouton sections of each genotype were used for quantification. For calculating the number of synaptic vesicles docked at the active zones, only those vesicles that were touching the T-bar platform were counted. For calculating the SSR thickness, the scale bar in the images was first calibrated to the number of pixels using the Set scale function in ImageJ/Fiji software. This was followed by using the Straight-line tool to draw a line across the SSR and Measure tool to calculate the thickness.

### Western blot analysis

The western blot analysis was done as previously described previouusly (Choudhury et al., 2016). Briefly, VNC from wandering third instar larvae were dissected out in ice-cold HL3 buffer and homogenized in buffer containing 50 mm Tris-HCl, pH 6.8, 25 mm KCl, 2 mm EDTA, 0.3 m sucrose, and 2% SDS in water. The homogenized sample was then mixed with an equal volume of 2× Laemmli buffer. The protein equivalent to 50 μg was separated on 12% SDS-PAGE and transferred to Hybond-LFP PVDF membrane (GE Healthcare, GE Healthcare Life Sciences). The membrane was then blocked with 5% skimmed milk for 1 h, followed by overnight incubation with anti-Rab11 (1:2000) and anti-Tubulin (1:5000) antibody. IRDye 800 (1:10 000) was used as a secondary antibody, and signals were visualized on LI-COR Odyssey platform. The density of Western bands was quantified using ImageJ/Fiji software.

### Quantification and statistical analysis

For fluorescence quantification, images were captured using a laser scanning confocal microscope (LSM780; Carl Zeiss or FV3000, Olympus). All the control and experimental fillets were processed in the same way, and the fluorescence images were captured under the same settings for every experimental set. For bouton quantification, CSP-labeled structures were counted at muscle 6/7 of A2 hemisegment. The number of boutons from each NMJ was normalized to the respective muscle area. To calculate the bouton number, NMJs from A2 hemisegment were captured using a plan apochromat 40× objective, 1.4 NA and all the CSP positive boutons were counted manually in ImageJ/Fiji software. For muscle area quantification, images from A2 hemisegment were captured using 20× objective, and the area was quantified using ZEN2 software (Carl Zeiss, Germany). For bouton number quantification, the total number of boutons per NMJ was divided by the respective muscle areas. For fluorescence intensity quantification, NMJs from muscle four were captured using a plan apochromat 60×, 1.4 NA objective. For each NMJ, the fluorescence intensity from each bouton was subtracted from the background intensity, and the average intensity was normalized to the control. The fluorescence intensity was calculated using ImageJ/Fiji software. For bouton area quantification, NMJs from muscle 6/7 at A2 hemisegment were captured, and the area was calculated by drawing a free-hand sketch around CSP positive bouton using ImageJ/Fiji software. For multiple comparisons, one-way ANOVA followed by *post hoc* Tukey’s test, or Student’s *t* test was used. GraphPad Prism 8 was used to plot the graph. Error bars in all the histograms represent SEM. *p <* *0.5, **p <* *0.01, ***p <* *0.001.

## Results

### *σ2-adaptin* genetically interacts with regulators of BMP signaling

**Figure 1.**
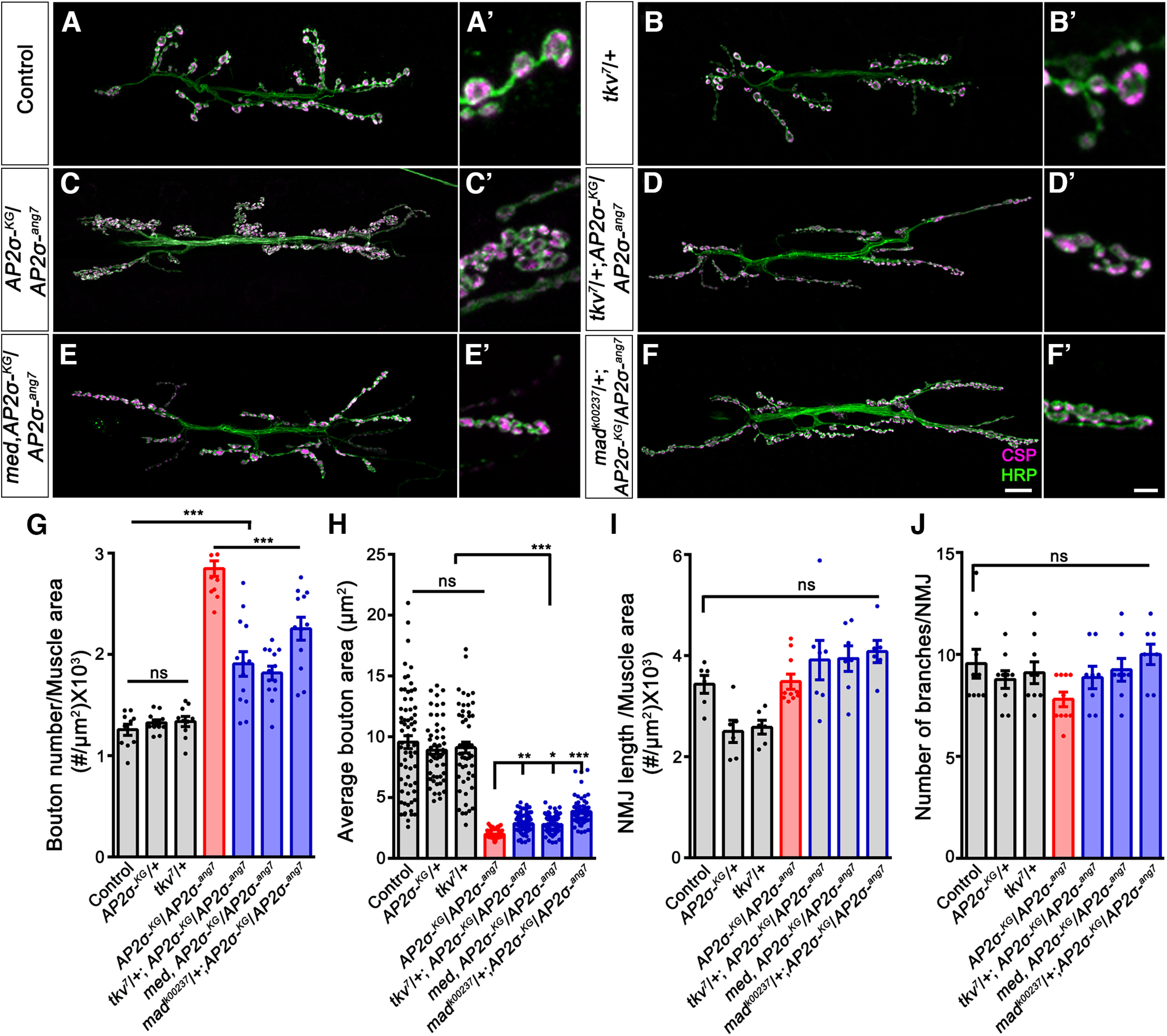
Downregulating BMP signaling components reduced the synaptic overgrowth and clustering in the *σ2-adaptin* mutant. ***A–F′***, Confocal images of NMJ synapses at muscles 6/7 of A2 hemisegment showing synaptic growth in (***A***, ***A′***) *w^1118^* (Control), (***B***, ***B′***) *tkv^7^*/+, (***C***, ***C′***) *AP2σ^KG02457^/AP2σ^ang7^*, (***D***, ***D′***) *tkv^7^*/+; *AP2σ^KG02457^/AP2σ^ang7^*, (***E***, ***E′***) *Med^13^*, *AP2σ^KG02457^/AP2σ^ang7^*, and (***F***, ***F′***) *Mad^k00237^*/+; *AP2σ^KG02457^/AP2σ^ang7^* double immunolabeled with a presynaptic vesicle marker, CSP (magenta), and a neuronal membrane marker, HRP (green), to mark the bouton outline. Reducing the levels of BMP signaling components in the *AP2σ^KG02457^/AP2σ^ang7^* background reduces the synaptic overgrowth. Scale bar in ***F*** (for ***A–F***) and ***F′*** (for ***A′–F′***) represents 20 and 5 μm, respectively. ***G***, Histogram showing the average bouton number normalized to the muscle area from muscle 6/7 NMJ at A2 hemisegment in control animals (1.26 ± 0.06), *AP2σ^KG02457^*/+ (1.33 ± 0.03), *tkv^7^*/+ (1.34 ± 0.05), *AP2σ^KG02457^/AP2σ^ang7^* (2.85 ± 0.08), *tkv^7^*/+; *AP2σ^KG02457^/AP2σ^ang7^* (1.91 ± 0.12), *Med^13^*, *AP2σ^KG02457^/AP2σ^ang7^* (1.82 ± 0.07), and *Mad^k00237^*/+; *AP2σ^KG02457^/AP2σ^ang7^* (2.25 ± 0.11). Error bar represents SEM; the statistical analysis was done using one-way ANOVA followed by *post hoc* Tukey’s test. ***H***, Histogram showing the average bouton area from muscle 6/7 NMJ at A2 hemisegment in control animals (9.57 ± 0.53), *AP2σ^KG02457^*/+ (8.89 ± 0.35), *tkv^7^*/+ (9.11 ± 0.46), *AP2σ^KG02457^/AP2σ^ang7^* (1.98 ± 0.03), *tkv^7^*/+; *AP2σ^KG02457^/AP2σ^ang7^* (2.85 ± 0.05), *Med^13^*, *AP2σ^KG02457^/AP2σ^ang7^* (2.79 ± 0.05), and *Mad^k00237^*/+; *AP2σ^KG02457^/AP2σ^ang7^* (3.86 ± 0.14). Error bar represents SEM; the statistical analysis was done using one-way ANOVA followed by *post hoc* Tukey’s test. *p < 0.05, **p < 0.01, ***p < 0.001; ns, not significant. ***I***, Histogram showing the average NMJ length normalized with muscle area from muscle 6/7 NMJ at A2 hemisegment in control animals (3.43 ± 0.18), *AP2σ^KG02457^*/+ (2.5 ± 0.22), *tkv^7^*/+ (2.5 ± 0.14), *AP2σ^KG02457^/AP2σ^ang7^* (3.49 ± 0.15), *tkv^7^*/+; *AP2σ^KG02457^/AP2σ^ang7^* (3.91 ± 0.39), *Med^13^*, *AP2σ^KG02457^/AP2σ^ang7^* (3.95 ± 0.25), and *Mad^k00237^*/+; *AP2σ^KG02457^/AP2σ^ang7^* (4.08 ± 0.22). Error bar represents SEM; the statistical analysis was done using one-way ANOVA followed by *post hoc* Tukey’s test. ns, not significant. ***J***, Histogram showing the average number of branches from muscle 6/7 NMJ at A2 hemisegment in control animals (9.56 ± 0.71), *AP2σ^KG02457^*/+ (8.77 ± 0.43), *tkv^7^*/+ (9.11 ± 0.53), *AP2σ^KG02457^/AP2σ^ang7^* (7.8 ± 0.36), *tkv^7^*/+; *AP2σ^KG02457^/AP2σ^ang7^* (8.88 ± 0.55), *Med^13^*, *AP2σ^KG02457^/AP2σ^ang7^* (9.25 ± 0.56), and *Mad^k00237^*/+; *AP2σ^KG02457^/AP2σ^ang7^* (10 ± 0.5). Error bar represents SEM; the statistical analysis was done using one-way ANOVA followed by *post hoc* Tukey’s test. ns, not significant. Data supporting the rescue of *σ2-adaptin* mutant phenotypes by reducing Wit receptors is shown in Extended Data Figure 1-1.

In a previous study, we showed *σ2-adaptin* mutants caused NMJ overgrowth and upregulation of pMad, an effector of the BMP pathway. Two previously characterized *σ2-adaptin* alleles, *AP2σ^ang7^,* a strong hypomorph and *AP2σ^KG02457^*, a null allele was used in the current study (Choudhury et al., 2016). To explore the role σ2-adaptin in regulating BMP signaling at the NMJ, we first assessed the epistatic interaction between *σ2-adaptin* and components of the BMP-signaling pathway. We found that introducing one mutant copy of *tkv*, *Med*, or *Mad* in *σ2-adaptin* mutant background significantly suppressed the synaptic overgrowth phenotype in these animals (Fig. 1*A–F*). The number of boutons normalized to the muscle area was significantly rescued in *tkv^7^*/+; *AP2σ^KG02457^/AP2σ^ang7^* (1.91 ± 0.12, *p* ≤ 0.001), *Med^13^, AP2σ^KG02457^/AP2σ^ang7^* (1.82 ± 0.07, *p* ≤ 0.001), and *Mad^k00237^*/+; *AP2σ^KG02457^/AP2σ^ang7^* (2.25 ± 0.11, *p* ≤ 0.001) when compared with *AP2σ^KG02457^*/*AP2σ^ang7^* (2.85 ± 0.08, *p* ≤ 0.001). However, there was no significant difference between *w^1118^* control, heterozygous *AP2σ^KG02457^*/+, and *tkv^7^*/+ (*w^1118^*: 1.26 ± 0.06, *AP2σ^KG02457^*/+: 1.33 ± 0.03 and *tkv^7^*/+: 1.34 ± 0.05; Fig. 1*G*).

10.1523/ENEURO.0044-22.2022.f1-1 Extended Data Figure 1-1 Reducing levels of Wit receptor rescues the bouton number in *σ2-adaptin* mutant. ***A–C***, Confocal images of NMJ synapses at muscles 6/7 NMJ at A2 hemisegment showing the synaptic growth in control (***A***), *AP2σ^KG02457^/AP2σ^ang7^* (***B***), *wit^A12^*, *AP2σ^KG02457^/AP2σ^ang7^* (***C***), double immunolabeled with a presynaptic vesicle marker, CSP (magenta), and a neuronal membrane marker, HRP (green), to mark the bouton outline. Reducing the levels of BMP Type II receptor in the *AP2σ^KG02457^/AP2σ^ang7^* background reduces the synaptic overgrowth. Scale bar in ***C*** represents 10 μm. ***D***, Histogram showing the average bouton number normalized to the muscle area from muscle 6/7 NMJ at A2 hemisegment in control animals (1.33 ± 0.03), *AP2σ^KG02457^/AP2σ^ang7^* (2.50 ± 0.11), and *wit^A12^*, *AP2σ^KG02457^/AP2σ^ang7^* (2.0 ± 0.07). Error bar represents SEM; the statistical analysis was done using one-way ANOVA followed by *post hoc* Tukey’s test. **p < 0.01, ***p < 0.001. Download Figure 1-1, TIF file.

Consistent with the above observations, we found that mutating one copy of *wit* significantly reduced the *σ2-adaptin* induced synaptic overgrowth (*wit^A12^, AP2σ^KG02457^/AP2σ^ang7^*: 2.00 ± 0.08 vs *AP2σ^KG02457^/AP2σ^ang7^*: 2.50 ± 0.11; *p* ≤ 0.01; Extended Data Fig. 1-1). Since elevated BMP signaling results in the formation of smaller boutons, we quantified the bouton area in these genotypes. We found that introducing one copy of *tkv^7^* (*tkv^7^*/+; *AP2σ^KG02457^/AP2σ^ang7^*: 2.9 ± 0.05, *p* ≤ 0.01), *Med^13^* (*Med^13^, AP2σ^KG02457^/AP2σ^ang7^*: 2.79 ± 0.05, *p* ≤ 0.05), and *Mad^k00237^*/+; *AP2σ^KG02457^/AP2σ^ang7^* (3.86 ± 0.15, *p* ≤ 0.001) in *σ2-adaptin* mutant background partially restored the bouton area when compared with *σ2-adaptin* mutant alone (*AP2σ^KG02457^/AP2σ^ang7^*:1.98 ± 0.03; *w^1118^* controls: 9.5 ± 0.53; Fig. 1*A′–F′*,*H*). Compared with *w^1118^* controls, no significant difference was found in the branching and length of the NMJ in these genotypes (Fig. 1*I*,*J*). Thus, our data suggest that σ2-adaptin genetically interacts with the BMP receptors to regulate NMJ bouton number and area.

**Figure 2.**
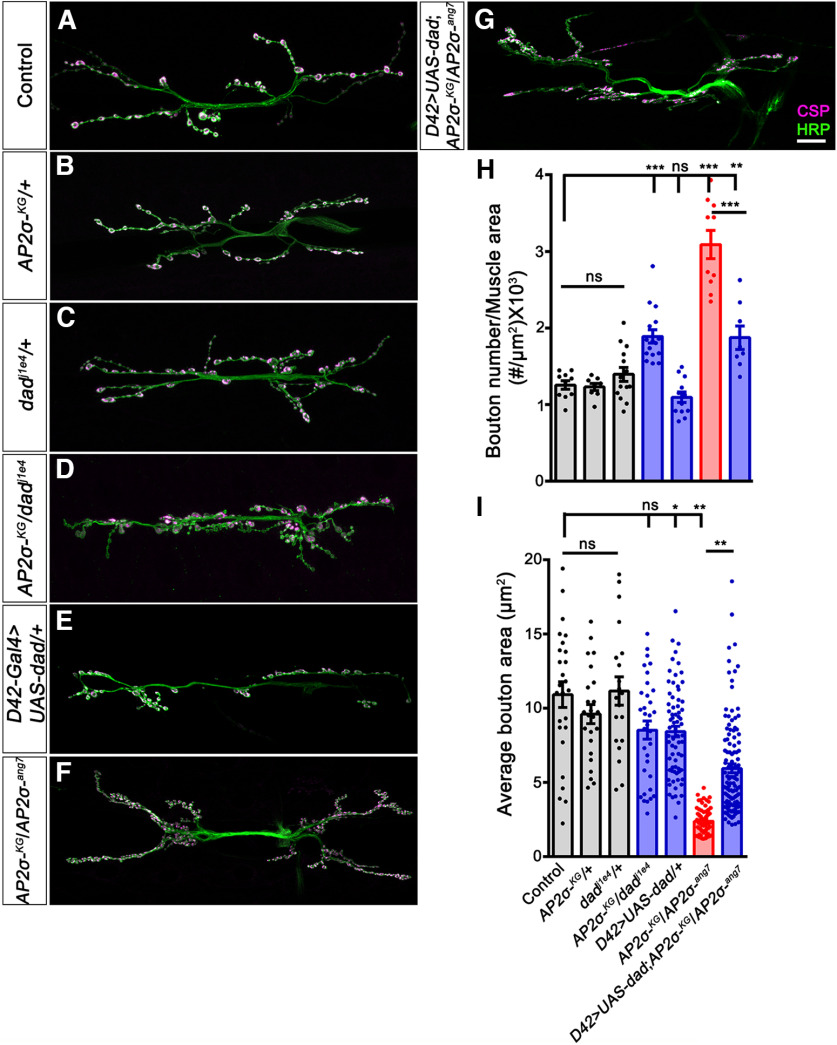
*σ2-adaptin* genetically interacts with *Dad*, the inhibitory Smad of BMP signaling. ***A–G***, Confocal images of NMJ synapses at muscle 6/7 NMJ at A2 hemisegment showing synaptic growth in (***A***) Control, (***B***) *AP2σ^KG02457^/+*, (***C***) *Dad^j1E4^/+*, (***D***) *AP2σ^KG02457^/Dad^j1E4^*, (***E***) *UAS-Dad/+; D42-Gal4/+*, (***F***) *AP2σ^KG02457^/AP2σ^ang7^*, and (***G***) *UAS-Dad/+*; *D42-Gal4, AP2σ^ang7^/AP2σ^KG02457^* double immunolabeled with a presynaptic synaptic vesicle marker, CSP (magenta), and a neuronal membrane marker, HRP (green), to mark the bouton outline. Note that trans-heterozygotes of *σ2-adaptin* and *Dad* mutants show significantly increased synaptic growth. Scale bar in ***G*** represents 20 μm. ***H***, Histogram showing average bouton number normalized to the muscle area from muscle 6/7 NMJ at A2 hemisegment in control animals (1.26 ± 0.06), *AP2σ^KG02457^*/+ (1.23 ± 0.05), *Dad^j1E4^*/+ (1.39 ± 0.09), *AP2σ^KG02457^/Dad^j1E4^* (1.89 ± 0.09), *UAS-Dad/+; D42-Gal4/+* (1.1 ± 0.07), *AP2σ^KG02457^/AP2σ^ang7^* (3.1 ± 0.18), and *UAS-Dad/+*; *D42-Gal4, AP2σ^ang7^/AP2σ^KG02457^* (1.87 ± 0.15). Error bar represents SEM; statistical analysis was done using one-way ANOVA followed by *post hoc* Tukey’s test. ***p <* *0.001; ns, not significant. ***I***, Histogram showing average bouton area from muscle 6/7 NMJ at A2 hemisegment in control animals (10.91 ± 0.87), *AP2σ^KG02457^*/+ (9.6 ± 0.63), *Dad^j1E4^*/+ (11.16 ± 0.95), *AP2σ^KG02457^/Dad^j1E4^* (8.5 ± 0.61), *UAS-Dad/+; D42-Gal4/+* (8.42 ± 0.36), *AP2σ^KG02457^/AP2σ^ang7^* (2.38 ± 0.11), and UAS*-Dad/+*; *D42-Gal4, AP2σ^ang7^/AP2σ^KG02457^* (5.94 ± 0.3). Error bar represents SEM; statistical analysis was done using one-way ANOVA followed by *post hoc* Tukey’s test. *p <* *0.05, **p <* *0.01; ns, not significant.

To further strengthen our observation that σ2-adaptin attenuates BMP signaling, we tested the interaction between σ2-adaptin and Dad, a negative regulator of BMP signaling (O’Connor-Giles et al., 2008; L Zhao et al., 2013). We first examined the total number of synaptic boutons in transheterozygotes of *σ2-adaptin* and *Dad* mutants (Fig. 2). While the number of boutons in larvae heterozygous for *σ2-adaptin* [*AP2σ^KG02457^*/+ (1.23 ± 0.05)] and *Dad* [*Dad^j1E4^/+* (1.39 ± 0.09)] was comparable with *w^1118^* control (1.26 ± 0.06), transheterozygous *Dad^j1E4^/AP2σ^KG02457^* (1.89 ± 0.09, *p* ≤ 0.001) showed significantly higher bouton number when compared with *w^1118^* controls (Fig. 2*H*). However, there was no significant difference in the bouton area in transheterozygotes of *σ2-adaptin* and *Dad* mutants *Dad^j1E4^/AP2σ^KG02457^* (8.51 ± 0.61) when compared with *w^1118^* control (10.91 ± 0.87; Fig. 2*I*).

Next, we examined whether neuronally expressing *UAS-Dad* in *σ2-adaptin* mutant could rescue the synaptic overgrowth phenotype (Fig. 2*E*,*F*). Neuronal expression of Dad in *σ2-adaptin* mutant background *UAS-Dad/+*; *D42-Gal4, AP2σ^ang7^/AP2σ^KG02457^* (1.87 ± 0.15, *p* ≤ 0.001) significantly rescued the synaptic overgrowth in *σ2-adaptin* mutant *AP2σ^ang7^/AP2σ^KG02457^* (3.1 ± 0.18, *p* ≤ 0.001; Fig. 2*G*). Moreover, we found that neuronal expression of Dad in *σ2-adaptin* mutant background partially rescued the average bouton area (*UAS-Dad/+*; *D42-Gal4, AP2σ^ang7^/AP2σ^KG02457^*: 5.94 ± 0.29, *p* ≤ 0.001; Fig. 2*I*). Taken together, these data suggest that σ2-adaptin regulates BMP signaling to attenuate synaptic growth at the *Drosophila* NMJ.

### The functional and morphologic aspects of σ2-adaptin can be genetically discriminated

**Figure 3.**
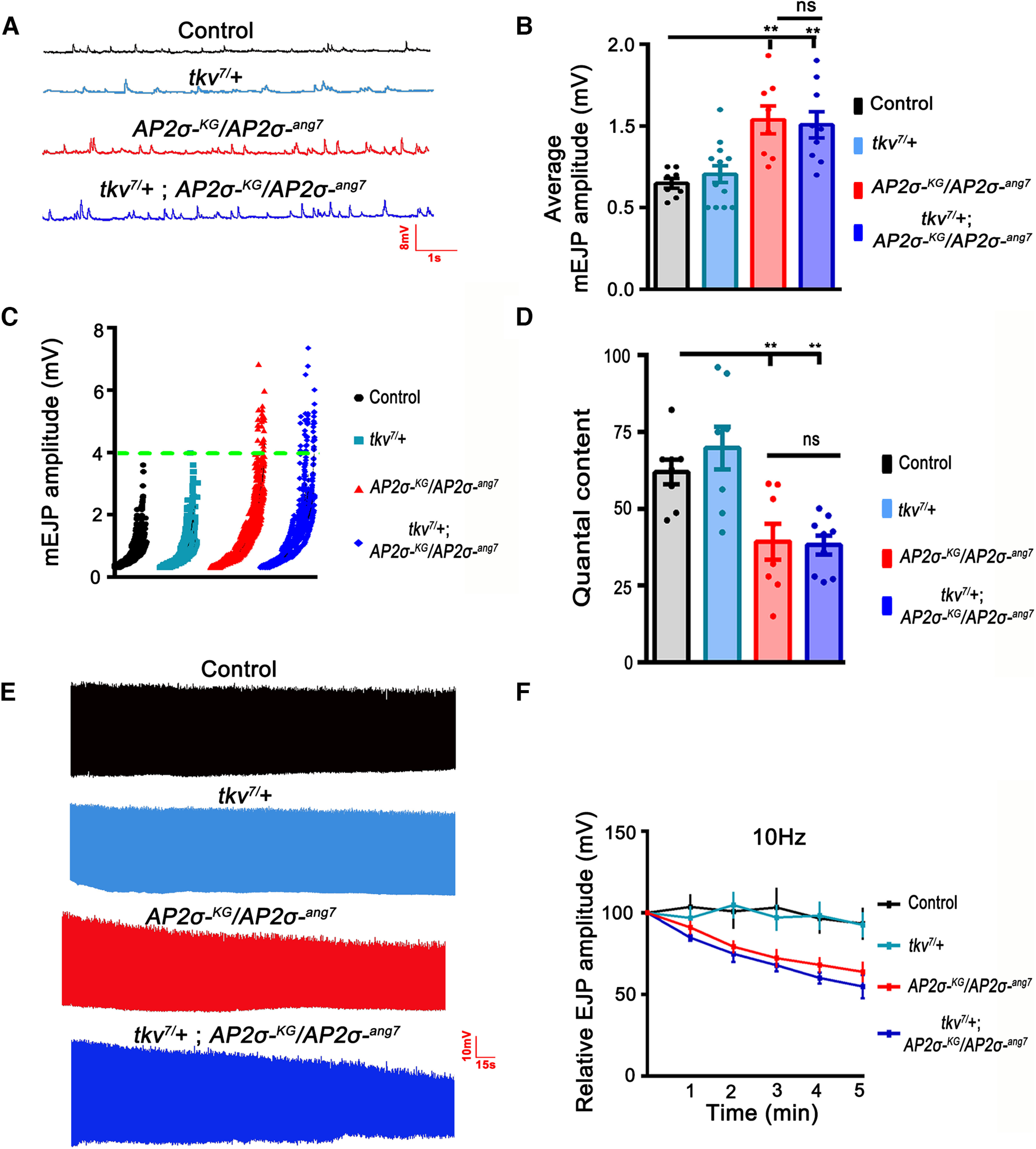
Structural and functional deficits in *σ2-adaptin* mutant can be genetically discriminated. ***A***, Representative traces of mEJP in control, *tkv^7^*/+, heteroallelic *AP2σ^KG02457^/AP2σ^ang7^*, and *tkv^7^*/+; *AP2σ^KG02457^/AP2σ^ang7^* larvae. ***B***, Quantification of average mEJP amplitude in control (0.65 ± 0.03), *tkv^7^*/+ (0.71 ± 0.05), heteroallelic *AP2σ^KG02457^/AP2σ^ang7^* (1.04 ± 0.09) and *tkv^7^*/+; *AP2σ^KG02457^/AP2σ^ang7^* (1.01 ± 0.08). At least eight NMJ recordings of each genotype were used for quantification. Error bars represent the SEM; statistical analysis is based on one-way ANOVA followed by *post hoc* Tukey’s multiple-comparison test. **p < 0.01; ns, not significant. ***C***, Scatter plot shows quantification of mEJP amplitude distribution in control, *tkv^7^*/+, heteroallelic *AP2σ^KG02457^/AP2σ^ang7^* and *tkv^7^*/+; *AP2σ^KG02457^/AP2σ^ang7^*. Note that events with mEJPs greater than 4 mV occurred more frequently in *AP2σ^KG02457^/AP2σ^ang7^* and *tkv^7^*/+; *AP2σ^KG02457^/AP2σ^ang7^* compared with controls. ***D***, Quantification of quantal content in control (61.92 ± 4.02), *tkv^7^*/+ (69.77 ± 6.98), *AP2σ^KG02457^/AP2σ^ang7^* (39.29 ± 5.81) and *tkv^7^*/+; *AP2σ^KG02457^/AP2σ^ang7^* (38.2 ± 3.09). At least eight NMJ recordings of each genotype were used for quantification. Error bars represent the SEM; statistical analysis is based on one-way ANOVA followed by *post hoc* Tukey’s multiple-comparison test. **p < 0.01; ns, not significant. ***E***, Representative traces of EJPs under high-frequency stimulation of control, *tkv^7^*/+, heteroallelic *AP2σ^KG02457^/AP2σ^ang7^* and *tkv^7^*/+; *AP2σ^KG02457^/AP2σ^ang7^* larvae stimulated at 10 Hz for 5 min in 1.5 mm Ca^2+^ containing HL3. ***F***, Line plot shows quantification of EJP amplitude during 10-Hz high-frequency recordings in control, *tkv^7^*/+, *AP2σ^KG02457^/AP2σ^ang7,^* and *tkv^7^*/+; *AP2σ^KG02457^/AP2σ^ang7^*. Note that the decline in EJP amplitude in *σ2-adaptin* mutant could not be rescued by reducing the levels of Tkv.

Since one mutant copy of BMP receptors in the *σ2-adaptin* mutant background significantly restores the morphologic defects, we asked whether the electrophysiological defects associated with the *σ2-adaptin* mutant could also be rescued. To address this, we measured evoked excitatory junction potential (EJP), quantal content (QC), and high-frequency intracellular recording on *w^1118^* control, heterozygous *tkv^7^/+*, *AP2σ^KG02457^/AP2σ^ang7^*, and *tkv^7^/+; AP2σ^KG02457^/AP2σ^ang7^* larvae (Fig. 3). We found that both mEJP amplitude and quantal content in *tkv^7^/+; AP2σ^KG02457^/AP2σ^ang7^* (mEJP amplitude = 1.01 ± 0.08, QC = 38.20 ± 3.09) were not significantly different from *σ2-adaptin* mutant *AP2σ^KG02457^/AP2σ^ang7^* (mEJP = 1.04 ± 0.09, QC = 39.29 ± 5.81; Fig. 3*A–D*). The scatter plot for mEJP amplitude distribution showed that both *σ2-adaptin* mutant and *tkv^7^/+; AP2σ^KG02457^/AP2σ^ang7^* had more mEJPs with higher amplitude compared with *w^1118^* controls (Fig. 3*C*). Furthermore, we found that reducing the levels of Tkv receptor did not rescue the activity-dependent decline in EJP amplitude. The line plot showed that both *σ2-adaptin* mutant and *tkv^7^/+; AP2σ^KG02457^/AP2σ^ang7^* followed the same trend of EJP amplitude decline during the high frequency (10 Hz) stimulations (Fig. 3*E*,*F*). Moreover, there was no significant difference between *w^1118^* and *tkv^7^*/+ controls in mEJP amplitude, QC, and high frequency recordings (*w^1118^*: mEJP = 0.65 ± 0.03, QC = 61.92 ± 4.02; *tkv^7^*/+: mEJP = 0.71 ± 0.05, QC = 69.77 ± 6.98; Fig. 3*B*,*D*,*F*). These data suggest that while reducing BMP signaling by lowering Tkv receptors in *σ2-adaptin* mutant significantly rescued neuronal overgrowth, it did not restore the physiological deficiencies in *σ2-adaptin* mutants indicating that functional and morphologic defects in *σ2-adaptin* mutants could be independent of one another.

### Loss of *σ2-adaptin* leads to the accumulation of endosome-like structures at the NMJ

**Figure 4.**
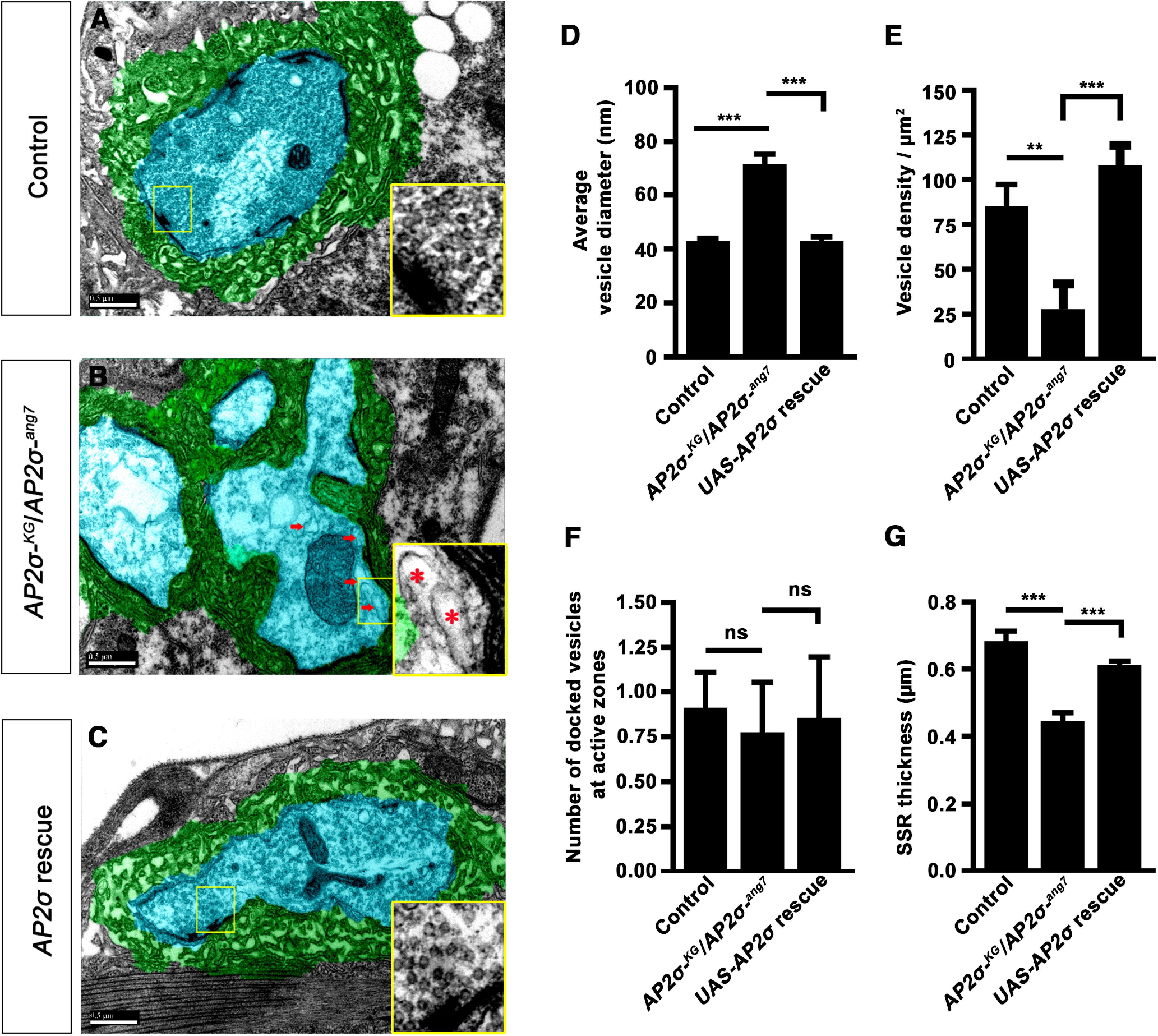
*σ2-adaptin* mutant synapses show accumulation of large endosome-like vesicular structure. ***A–C***, Electron micrographs of third instar Type Ib boutons of control (***A***), *AP2σ^KG02457^/AP2σ^ang7^* (***B***), and *actin5C*/+; *AP2σ^KG02457^*, *UAS-AP2σ*/*AP2σ^ang7^* (***C***). Arrows point to the large endosome-like vesicular structures observed in *AP2σ^KG02457^/AP2σ^ang7^* boutons but are absent in control and rescued boutons. The insets show a magnified area around the active zones. The presynaptic compartment is pseudocolored in cyan, and the subsynaptic reticulum is marked in green. Scale bar represents 500 nm. ***D***, Histogram showing average vesicle diameter in control (43.16 ± 0.94), *AP2σ^KG02457^/AP2σ^ang7^* (71.53 ± 3.7), and *actin5C*/+; *AP2σ^KG02457^*, *UAS-AP2σ*/*AP2σ^ang7^* (43.1 ± 1.46). Error bar represents SEM; statistical analysis was done using one-way ANOVA followed by *post hoc* Tukey’s test. ***p <* *0.001, **p < 0.01. ***E***, Histogram showing SV density per unit area in control (85.18 ± 12.26), *AP2σ^KG02457^/AP2σ^ang7^* (28.02 ± 14), and *actin5C*/+; *AP2σ^KG02457^*, *UAS-AP2σ*/*AP2σ^ang7^* (107.9 ± 11.32). At least 10 images from three different larvae per genotype were used for quantification. Error bar represents SEM; statistical analysis was done using one-way ANOVA followed by *post hoc* Tukey’s test. ***p <* *0.001, **p < 0.01. ***F***, Histogram showing average number of synaptic vesicles docked at the active zones in control (0.91 ± 0.20), *AP2σ^KG02457^/AP2σ^ang7^* (0.78 ± 0.28), and *actin5C*/+; *AP2σ^KG02457^*, *UAS-AP2σ*/*AP2σ^ang7^* (0.86 ± 0.34). At least five images from three different larvae per genotype were used for quantification. Error bar represents SEM; statistical analysis was done using one-way ANOVA followed by *post hoc* Tukey’s test. ns, not significant. ***G***, Histogram showing SSR thickness in control (0.68 ± 0.03), *AP2σ^KG02457^/AP2σ^ang7^* (0.45 ± 0.03), and *actin5C*/+; *AP2σ^KG02457^*, *UAS-AP2σ*/*AP2σ^ang7^* (0.61 ± 0.01). At least five images from three different larvae per genotype were used for quantification. Error bar represents SEM; statistical analysis was done using one-way ANOVA followed by *post hoc* Tukey’s test. ***p <* *0.001.

Studies have shown that internalization and recycling of the membrane and BMP receptors depend on CME and endosomal proteins such as Rab5 and Rab11 (Mitchell et al., 2004; Liu et al., 2014). Mutants with defects in endocytosis and endosomal trafficking cause functional and ultrastructural abnormalities at the synapses. To get a deeper understanding of how loss of σ2-adaptin affects synapse ultrastructure, we performed transmission electron microscopy on *σ2-adaptin* mutant NMJs. Interestingly, ultrastructural analysis of *σ2-adaptin* deficient synapses showed an accumulation of large endosome-like structures, similar to what has been shown in mutants that affect endocytic and endosomal recycling machinery such as clathrin (Kasprowicz et al., 2008; Kawasaki et al., 2011), AP180 (B Zhang et al., 1998), Rab5 (Shimizu et al., 2003), Rab8 (West et al., 2015), and Rab11 (Inoshita et al., 2017). We found decreased synaptic vesicle (SV) density (*w^1118^*: 85.18 ± 12.26 vs *AP2σ^KG02457^/AP2σ^ang7^*: 28.02 ± 14, *p* ≤ 0.01) and increased size of the SVs in the *σ2-adaptin* mutants (*w^1118^*: 43.16 ± 0.94 vs *AP2σ^KG02457^/AP2σ^ang7^*: 71.53 ± 3.70, *p* ≤ 0.001). While there was no change in the number of SVs docked at the active zones (*w^1118^*: 0.91 ± 0.20 vs *AP2σ^KG02457^/AP2σ^ang7^*: 0.78 ± 0.28, *p* > 0.05), we found significantly decreased thickness of the subsynaptic reticulum (SSR; *w^1118^*: 0.68 ± 0.03 vs *AP2σ^KG02457^/AP2σ^ang7^*: 0.45 ± 0.03, *p* ≤ 0.001) in the *σ2-adaptin* mutants. These ultrastructural defects were rescued on ubiquitous expression of a *σ2-adaptin* transgene [*actin5C-Gal4*/+; *AP2σ^KG02457^*, *UAS-AP2σ*/*AP2σ^ang7^*: SV density (107.9 ± 11.32), SV size (43.1 ± 1.46), and SSR thickness (0.61 ± 0.01); Fig. 4*A–G*]. Together, these data indicate that compromised regeneration of vesicles from the presynaptic membrane and defective membrane recycling in *σ2-adaptin* mutants results in the accumulation of large endosome-like structures.

### *σ2-adaptin* mutants display increased Tkv receptors at the NMJ

**Figure 5.**
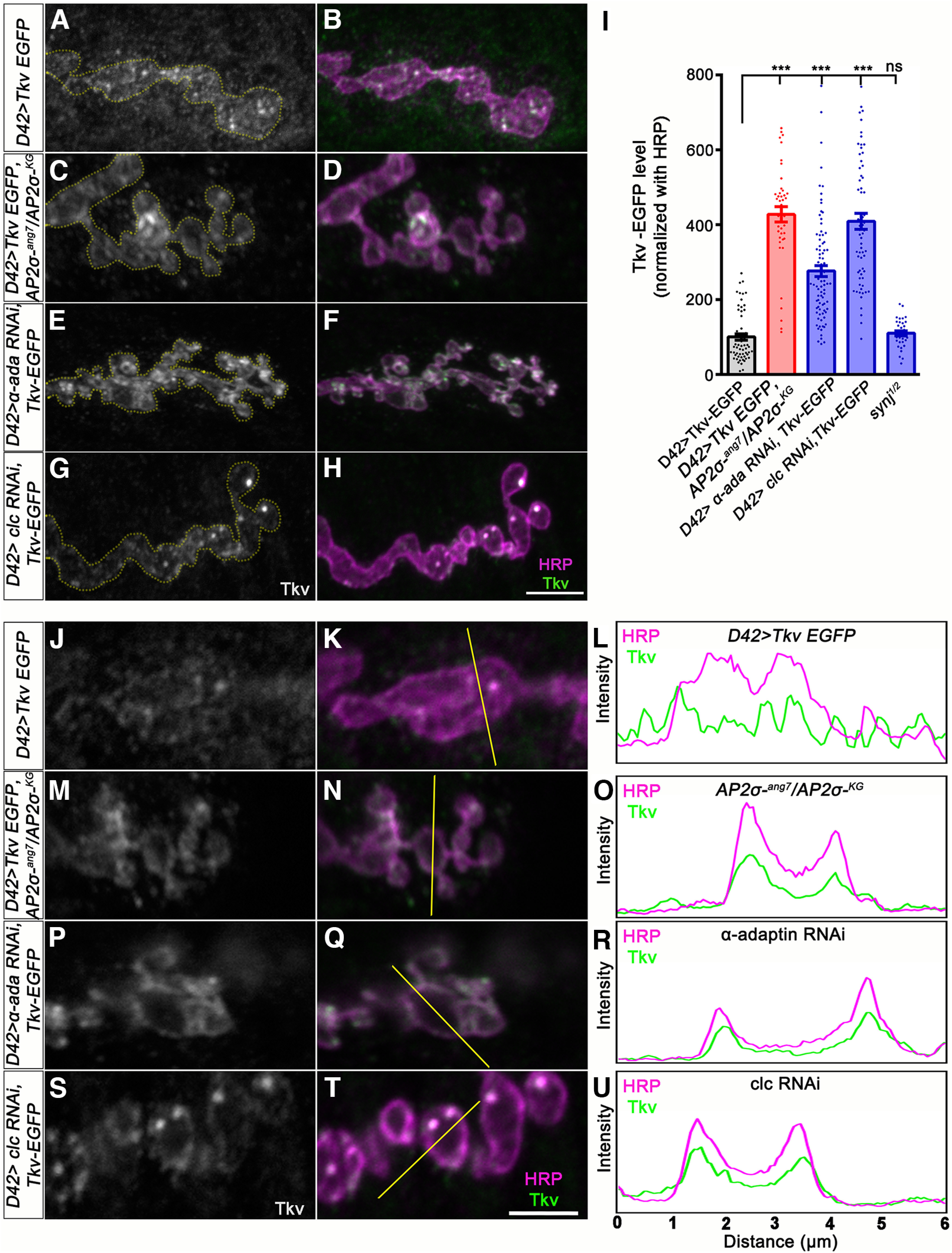
Neuronal reduction of subunits of AP2 complex or clathrin leads to accumulation of synaptic Tkv. ***A–H***, Confocal images of NMJ synapses at muscle 4 NMJ at A2 hemisegment in control, *D42-Gal4*/*UAS-tkv-EGFP* (***A***, ***B***), *D42-Gal4*, *AP2σ^KG02457^*/*AP2σ^ang7^*, *UAS-tkv-EGFP* (***C***, ***D***), *D42-Gal4, tkv-EGFP/*α*-adaptin* dsRNA (***E***, ***F***), and *D42-Gal4, tkv-EGFP/Clc* dsRNA (***G***, ***H***). The neuronal membrane is marked with HRP (magenta), and EGFP fluorescence is shown in grayscale/green. The bouton area is outlined in the gray channel. Scale bar in ***H*** represents 5 μm. ***I***, Histogram showing the relative Tkv level normalized to HRP in *D42-Gal4/UAS-tkv-EGFP* (100 ± 8.489), *D42-Gal4*, *AP2σ^KG02457^*/*AP2σ^ang7^*, *UAS-tkv-EGFP* (427.5 ± 20.2), *D42-Gal4, tkv-EGFP/*α*-adaptin* dsRNA (276.1 ± 14.75), *D42-Gal4, tkv-EGFP/Clc* dsRNA (408.6 ± 21.17), and *Synj^1/2^* (110.1 ± 6.65) synapses. Error bars represent SEM; statistical analysis is based on one-way ANOVA followed by *post hoc* Tukey’s multiple-comparison test. ***p < 0.001; ns, not significant. ***J–U***, A single confocal section of a bouton labeled for Tkv (represented in grayscale) and presynaptic membrane marker HRP (magenta) in *D42-Gal4*/*UAS-tkv-EGFP* (***J***, ***K***) or *D42-Gal4*, *AP2σ^KG02457^*/*AP2σ^ang7^*, *UAS-tkv-EGFP* (***M***, ***N***), *D42-Gal4, tkv-EGFP/α-adaptin* dsRNA (***P***, ***Q***), and *D42-Gal4, tkv-EGFP/Clc* dsRNA (***S***, ***T***). Note that the intensity profile plot across bouton (shown in ***L***, ***O***, ***R***, ***U*** as a thin line) shows that *σ2-adaptin* mutant (***O***), *D42-Gal4*>α*-adaptin* dsRNA (***R***), and *D42-Gal4>Clc* dsRNA (***U***) has more Tkv receptors at the membrane compared with control (***L***). Scale bar in ***T*** represents 2.5 μm.

AP2 complex has been shown to regulate CME and activity-dependent vesicle regeneration from endosome-like vacuoles (Kononenko et al., 2014; Kadlecova et al., 2017). Since the ultrastructural analysis of *σ2-adaptin* mutants revealed an accumulation of large endosome-like membranous structures similar to mutants with perturbed endocytosis or endosomal trafficking such as *Rab5*, *Rab8*, and *Rab11* mutants (Shimizu et al., 2003; West et al., 2015; Inoshita et al., 2017), we hypothesized that *σ2-adaptin* could be involved either in endocytosis of BMP receptors from the presynaptic membrane or in the endosome-dependent trafficking of the receptors. To test this possibility, we first assessed the level of Tkv receptors at the larval NMJ. Since specific antibodies against Tkv receptors are not available, we expressed an EGFP-tagged Tkv receptor transgene in the motor neurons of *σ2-adaptin* mutants. Interestingly, we found a significant accumulation of Tkv receptors at the mutant synapses (*D42-Gal4*, *AP2σ^KG02457^*/*AP2σ^ang7^*, *UAS-tkv-EGFP*: 427.5 ± 20.20, *p* ≤ 0.001) when compared with control (*D42-Gal4*/*UAS-tkv-EGFP*: 100 ± 8.49; Fig. 5*A–D*,*I*). In order to analyze the subcellular accumulation, we plotted the intensity profiles of Tkv-EGFP and HRP (a presynaptic membrane maker) in single sections of the acquired images. While in control synapses, Tkv localized both at the presynaptic membrane as well as within the bouton; we found a higher intensity peak of Tkv-EGFP at the presynaptic membranes of *σ2-adaptin* mutants (Fig. 5*J–O*). Consistent with these observations, we found that knocking down α-adaptin (*D42-Gal4*>*α-adaptin* dsRNA; 276.1 ± 14.75, *p* ≤ 0.001)] or clathrin light chain (Clc; *D42-Gal4*>*Clc* dsRNA; 408.61 ± 21.17, *p* ≤ 0.001) in motor neurons showed significantly increased synaptic Tkv levels (Fig. 5*E–I*) with intensity profiles similar to that of *σ2-adaptin* mutants (Fig. 5*P–U*). In contrast, mutants defective in proteins involved in later stages of endocytosis did not show Tkv accumulation at the presynaptic membrane (Fig. 5*I*), which is consistent with previous studies (O’Connor-Giles et al., 2008; G Zhao et al., 2015). These data indicate that endocytosis/trafficking of Tkv receptors in *σ2-adaptin* mutants is severely compromised, leading to their accumulation at the synaptic membranes.

### Loss of σ2-adaptin results in decreased levels of recycling endosomal marker Rab11

**Figure 6.**
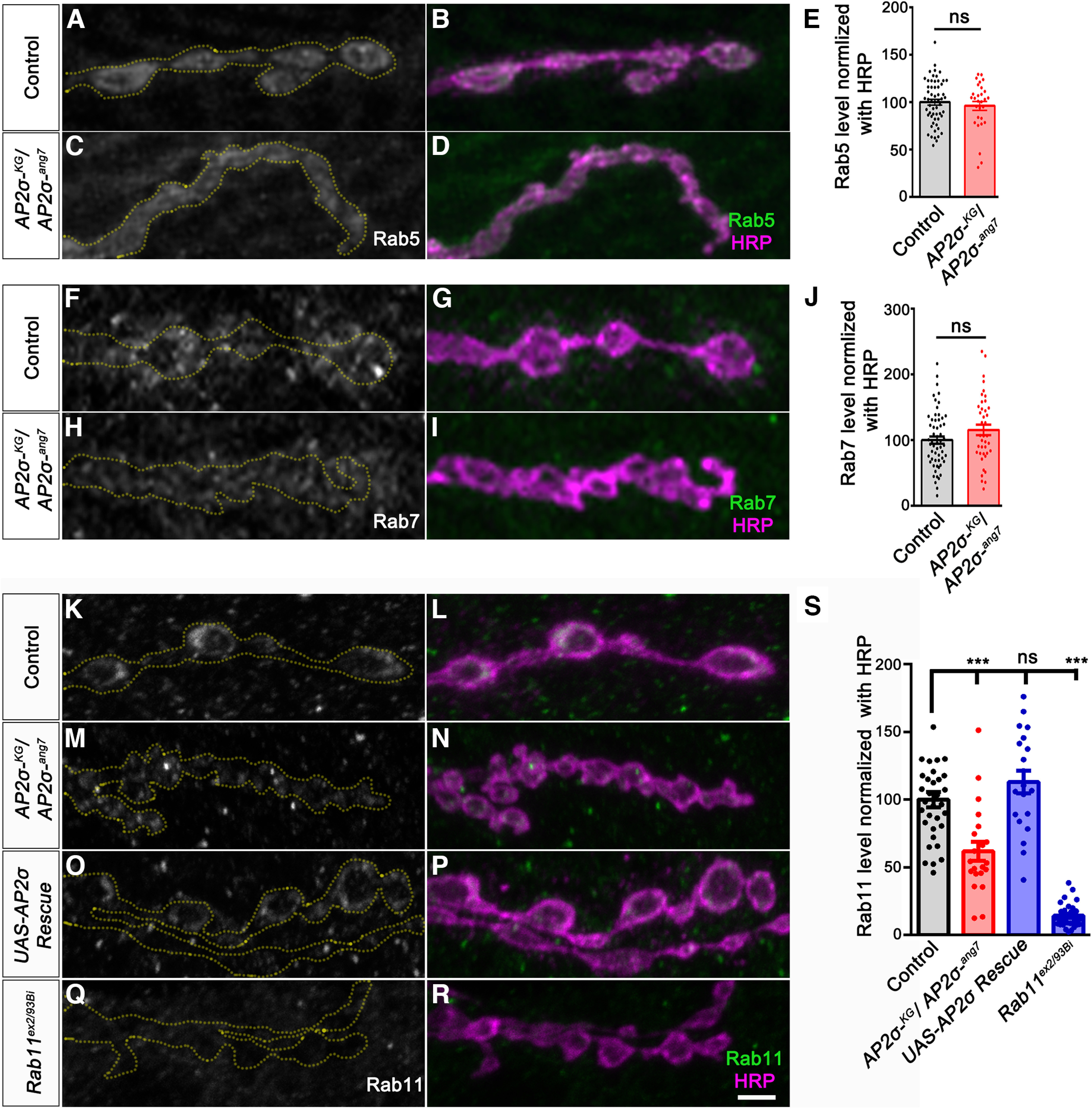
*σ2-adaptin* mutant synapses show a reduction in the recycling endosome marker, Rab11. ***A–D***, Confocal images of NMJ synapses at muscle 4 of A2 hemisegment in control (***A***, ***B***), *AP2σ^KG02457^/AP2σ^ang7^* (***C***, ***D***), double immunolabeled with early endosomal marker Rab5 (represented in grayscale/green) and neuronal membrane marker, HRP (magenta). ***E***, Histogram showing the Rab5 level in control (100.0 ± 3.0) and *AP2σ^KG02457^*/*AP2σ^ang7^* (97.25 ± 4.81) synapse. Error bar represents SEM; statistical analysis was done using Student’s *t* test. ns, not significant. ***F–I***, Confocal images of NMJ synapses at muscle 4 of A2 hemisegment in control (***F***, ***G***), *AP2σ^KG02457^/AP2σ^ang7^* (***H***, ***I***), double immunolabeled with late endosomal marker, Rab7 (represented in grayscale/green), and neuronal membrane marker, HRP (magenta). ***J***, Histogram showing the Rab7 level in control (100.0 ± 5.71) and *AP2σ^KG02457^*/*AP2σ^ang7^* (115.6 ± 8.17) synapse. Error bar represents SEM; statistical analysis was done using Student’s *t* test. ns, not significant. ***K–R***, Confocal images of NMJ synapses at muscle 4 of A2 hemisegment in control (***K***, ***L***), *AP2σ^KG02457^/AP2σ^ang7^* (***M***, ***N***), *D42-Gal4*, *AP2σ^KG02457^*/*UAS-AP2σ*, *AP2σ^ang7^* (***O***, ***P***), and *Rab11^ex2/93 Bi^* (***Q***, ***R***) double immunolabeled with recycling endosomal marker, Rab11 (represented in grayscale/green), and neuronal membrane marker, HRP (magenta). Scale bar in ***R*** represents 3 μm. ***S***, Histogram showing relative Rab11 level normalized to HRP in control (100 ± 5.73), *AP2σ^KG02457^*/*AP2σ^ang7^* (61.81 ± 7.11); *D42-Gal4*, *AP2σ^KG02457^*/*UAS-AP2σ*, *AP2σ^ang7^* (112.9 ± 8.29) and *Rab11^ex2/93 Bi^* (14.21 ± 1.57) synapses. Error bars represent SEM; statistical analysis is based on one-way ANOVA followed by *post hoc* Tukey’s multiple-comparison test. ***p < 0.001; ns, not significant. The data supporting that the levels of Rab11 is not altered in *σ2-adaptin* mutants is provided in Extended Data Figure 6-1.

Receptors are known to be endocytosed, trafficked to early endosomes, and sorted out for recycling back to the membrane or degraded in the lysosomes (Dunn et al., 1989; Gruenberg et al., 1989; Jovic et al., 2010). This process is mediated by the Rab family of small GTPases (Schwartz et al., 2007). Since the ultrastructural analysis of *σ2-adaptin* mutant synapse showed an accumulation of enlarged synaptic vesicles similar to mutants of the endolysosomal pathway (Shimizu et al., 2003; West et al., 2015; Inoshita et al., 2017), we next asked whether this pathway has any role in the accumulation of Tkv in *σ2-adaptin* mutants. We found that the synaptic levels of the early endosomal marker, Rab5 (*w^1118^*: 100.0 ± 3.0 vs *AP2σ^KG02457^/AP2σ^ang7^*: 97.25 ± 4.81) or the late endosomal marker, Rab7 (*w^1118^*: 100.0 ± 5.71 vs *AP2σ^KG02457^/AP2σ^ang7^*: 115.6 ± 8.17) were not altered in *σ2-adaptin* mutant synapses (Fig. 6*A–J*). However, we observed reduced synaptic Rab11 levels (*w^1118^*: 100.0 ± 5.73 vs *AP2σ^KG02457^/AP2σ^ang7^*: 61.81 ± 7.10, *p* ≤ 0.001) in *σ2-adaptin* mutant synapses (Fig. 6*K–S*). Western blot analysis revealed that total levels of Rab11 protein in *σ2-adaptin* mutants were not altered compared with *w^1118^* controls (Extended Data Fig. 6-1). Synaptic Rab11 levels were restored to *w^1118^* control levels on neuronal expression of a *σ2-adaptin* transgene in the *σ2-adaptin* mutant (*D42-Gal4*, *AP2σ^KG02457^*/*UAS-AP2σ*, *AP2σ^ang7^*:112.9 ± 8.29; Figure 6*O*,*P*,*S*). Taken together, these data indicate that: (1) compromised endocytosis, and possible defective recycling of the endocytosed Tkv receptors result in their accumulation at the synapse, or/and (2) AP2 complex may regulate Tkv sorting possibly through the endosomal pathway.

10.1523/ENEURO.0044-22.2022.f6-1 Extended Data Figure 6-1 *σ2 adaptin* mutants have normal Rab11 protein levels. ***A***, Western blotting shows the levels of total Rab11 for control, *AP2σ^KG02457^/AP2σ^ang7^*, *D42-Gal4*, *AP2σ^ang7^*/*UAS- AP2σ, AP2σ^KG02457^*, and *Rab11^ex2/93Bi^*. Note that total Rab11 protein levels are identical across all genotypes except *Rab11^ex2/93Bi^*. ***B***, Histogram showing the average levels of Rab11 normalized with internal control tubulin for control (100 ± 4.65), *AP2σ^KG02457^/AP2σ^ang7^* (102.8 ± 11.07), *D42-Gal4*, *AP2σ^ang7^*/*UAS- AP2σ, AP2σ^KG02457^* (84.45 ± 6.11), and *Rab11^ex2/93Bi^* (65.06 ± 7.16). Error bar represents SEM; the statistical analysis was done using one-way ANOVA followed by *post hoc* Tukey’s test. *p <* *0.05; ns, not significant. Download Figure 6-1, TIF file.

### Rab11 mutants phenocopy BMP-signaling and NMJ defects of σ2-adaptin

**Figure 7.**
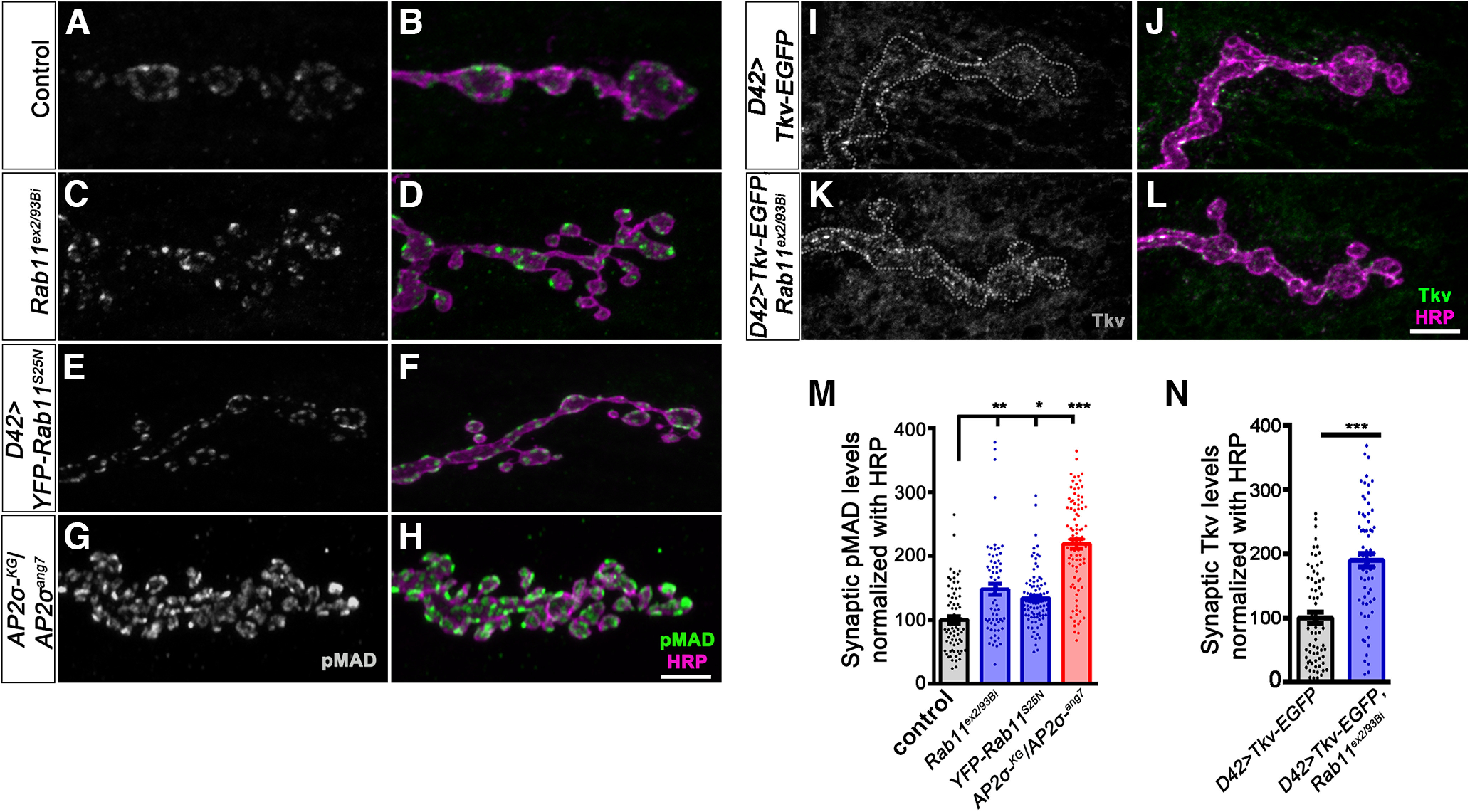
Rab11 mutants show elevated levels of synaptic pMad and Tkv. ***A–H***, Confocal images of NMJ from muscle 4 at A2 hemisegment in control (***A***, ***B***), *Rab11^ex2/93 Bi^* (***C***, ***D***), *D42-Gal4*-driven dominant-negative *YFP-Rab11^S25N^* (***E***, ***F***), and *AP2σ^KG02457^/AP2σ^ang7^* (***G***, ***H***) double immunolabeled with pMad (grayscale/green) and a neuronal membrane marker, HRP (magenta) to mark the bouton outline. Scale bar in ***H*** represents 5 μm. ***I–L***, Confocal images of NMJ from muscle 4 at A2 hemisegment in *D42-Gal4*-driven *tkv-EGFP* (***I***, ***J***) and *D42-Gal4*-driven *tkv-EGFP*, *Rab11^ex2^*/*Rab11^93Bi^* (***K***, ***L***) double immunolabeled with Tkv (grayscale/green) and a neuronal membrane marker, HRP (magenta) to mark the bouton outline. Scale bar in ***L*** represents 5 μm. ***M***, Histogram showing the levels of pMad normalized with HRP from muscle 4 at A2 hemisegment in control (100 ± 5.87), *Rab11^ex2/93 Bi^* (147.9 ± 8.58), *D42*-driven YFP-*Rab11^S25N^* (134.2 ± 4.36), and *AP2σ^KG02457^/AP2σ^ang7^* (218.8 ± 7.5). The error bar represents SEM; statistical analysis was done using one-way ANOVA followed by *post hoc* Tukey’s test. ***p <* *0.001, **p < 0.01, *p < 0.05. ***N***, Histogram showing the relative Tkv level normalized to HRP in *D42-Gal4-*driven *tkv-EGFP* (100 ± 8.71), and *D42-Gal4, Rab11^ex2^/tkv-EGFP, Rab11^93Bi^* (189.5 ± 10.57). Error bar represents SEM; statistical analysis was done using Student’s *t* test. ***p <* *0.001. The data supporting that the Tkv receptor is not enriched at the presynaptic membrane in *Rab11* mutants is shown in Extended Data Figure 7-1.

Rab11 has been shown to regulate BMP signaling at the *Drosophila* NMJ (Khodosh et al., 2006; Liu et al., 2014). Because *σ2-adaptin* mutant synapses showed reduced Rab11 levels, we next asked whether Rab11 had a role in regulating Tkv receptor trafficking. We first examined the levels of pMad and Tkv in *Rab11* mutants. We found that *Rab11* mutants, as well as animals expressing a dominant-negative form of Rab11 in motor neurons, showed accumulation of pMad at the NMJ synapses (*w^1118^*: 100 ± 5.87; *Rab11^ex2/93 Bi^*: 147.9 ± 8.58, *p* ≤ 0.01; *UAS-YFP-Rab11^S25N^*/+; *D42-Gal4/+*: 134.2 ± 4.36, *p* ≤ 0.05; *AP2σ^KG02457^/AP2σ^ang7^*: 218.8 ± 7.5, *p* ≤ 0.001; Fig. 7*A–H*,*M*). We further assessed Tkv receptor levels in these mutant synapses and found that *Rab11* mutants also showed increased Tkv levels at the synapse (*D42-Gal4, Rab11^ex2^/tkv-EGFP, Rab11^93Bi^*: 189.5 ± 10.57) compared with control animals (*D42-Gal4/tkv-EGFP*: 100 ± 8.71). However, the Tkv enrichment pattern was not the same as that in *σ2-adaptin* mutants (Fig. 7*I–L*,*N*). While *σ2-adaptin* mutants showed Tkv enrichment at the synaptic membranes, *Rab11* mutants had punctate Tkv localization within the boutons, but showed no enrichment at the presynaptic membrane (Extended Data Fig. 7-1). This suggests that while Rab11 does not affect the internalization of Tkv, a defective vesicular trafficking pathway in *Rab11* mutants probably leads to the enrichment of Tkv receptors in endosome-like compartments.

10.1523/ENEURO.0044-22.2022.f7-1 Extended Data Figure 7-1 Tkv-EGFP is not localized at the presynaptic membrane in the *Rab11* mutants. ***A–F***, A single confocal section of a bouton labelled for Tkv (represented in grayscale) and presynaptic membrane marker HRP (magenta) in *D42-Gal4*/*UAS-tkv-EGFP* (***A***, ***B***) and *D42-Gal4*/*UAS-tkv-EGFP* (***D***, ***E***). The intensity profile across the bouton in *D42-Gal4*/*UAS-tkv-EGFP* (***F***) suggests that Tkv is not enriched at the presynaptic membrane. Download Figure 7-1, TIF file.

**Figure 8.**
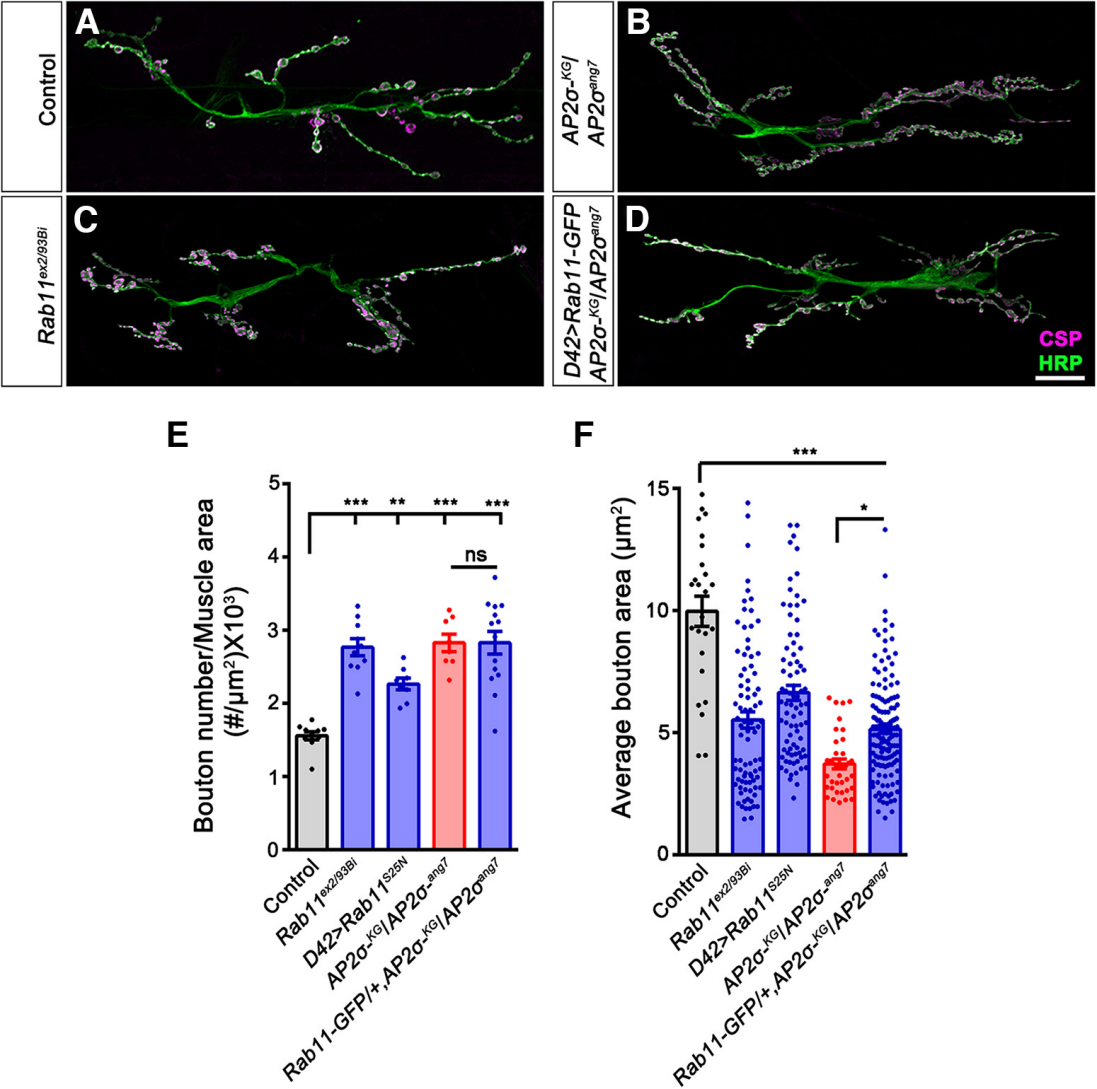
*Rab11* mutants phenocopy NMJ structural defects of *σ2-adaptin* mutation. ***A–D***, Confocal images of NMJ synapses at muscle 6/7 NMJ at A2 hemisegment in control (***A***), *AP2σ^KG02457^/AP2σ^ang7^* (***B***), *Rab11^ex2/93 Bi^* (***C***), and *D42-Gal4*>*YFP-Rab11^S25N^* (***D***) double immunolabeled with a presynaptic synaptic vesicle marker, CSP (magenta), and a neuronal membrane marker, HRP (green), to mark the bouton outline. Scale bar in ***D*** represents 20 μm. ***E***, Histogram showing average bouton number normalized to the muscle area from muscle 6/7 NMJ at A2 hemisegment in control (1.56 ± 0.06), *Rab11^ex2/93 Bi^* (2.77 ± 0.11), *D42-Gal4*-driven dominant-negative *YFP-Rab11^S25N^* (2.26 ± 0.08), AP*2σ^KG02457^/AP2σ^ang7^* (2.83 ± 0.12), and UAS*-YFP-Rab11^WT^*/+; *D42-Gal4*, *AP2σ^ang7/^AP2σ^KG02457^* (2.83 ± 0.16). Error bar represents SEM; statistical analysis was done using one-way ANOVA followed by *post hoc* Tukey’s test. ***p <* *0.001, *p <* *0.05; ns, not significant. ***F***, Histogram showing average bouton area from muscle 6/7 NMJ at A2 hemisegment in control (9.97 ± 0.62), *Rab11^ex2/93 Bi^* (5.51 ± 0.33), *D42-Gal4*-driven dominant-negative *YFP-Rab11^S25N^* (6.63 ± 0.31), AP*2σ^KG02457^/AP2σ^ang7^* (3.71 ± 0.2), and *UAS-YFP-Rab11^WT^*/+; *D42-Gal4*, *AP2σ^ang7/^AP2σ^KG02457^* (5.14 ± 0.17). Error bar represents SEM; statistical analysis was done using one-way ANOVA followed by *post hoc* Tukey’s test. ***p <* *0.001, *p <* *0.05; ns, not significant. Data supporting that expression of wild-type or constitutive active Rab11 does not alter NMJ morphology and does not restore NMJ morphologic defects of σ2-adaptin mutants is shown in Extended Data Figures 8-1 and 8-2, respectively. Data supporting that expression of wild-type or dominant negative Rab11 and constitutive active Rab11 does not restore the functional defects of *σ2-adaptin* mutants is shown in Extended Data Figures 8-3 and 8-4, respectively.

Since *σ2-adaptin* mutants showed reduced Rab11 levels at the NMJ, we next asked whether Rab11 was responsible for the morphologic defects in these mutants. To assess this, we first examined whether reducing Rab11 levels phenocopied *σ2-adaptin* mutations. Interestingly, we found that *Rab11* mutants or neuronal expression of a dominant-negative form of Rab11 (*Rab11^S25N^*) indeed phenocopied the NMJ morphologic defects in *σ2-adaptin* mutants and showed NMJ overgrowth (*w^1118^*: 1.56 ± 0.06; *Rab11^ex2/93 Bi^*: 2.77 ± 0.11, *p* ≤ 0.001; *UAS-YFP-Rab11^S25N^*/+; *D42-Gal4*/+: 2.26 ± 0.08, *p* ≤ 0.001; and *AP2σ^KG02457^/AP2σ^ang7^*: 2.83 ± 0.12, *p* ≤ 0.001; Fig. 8*A–F*). Expressing a wild-type or constitutively active form of Rab11, however, did not alter the synaptic morphology (Extended Data Fig. 8-1). We next examined whether neuronal expression of Rab11 could restore the synaptic overgrowth in *σ2-adaptin* mutants. We found that expressing the wild-type form of Rab11 in *σ2-adaptin* mutant background (*UAS-YFP-Rab11^WT^*/+; *D42-Gal4*, *AP2σ^ang7/^AP2σ^KG02457^*: 2.83 ± 0.16, ns) did not prevent the synaptic overgrowth (Fig. 8*E*,*F*). Moreover, expressing a constitutively active form of Rab11 (Rab11^CA^; *UAS-YFP-Rab11^CA^*/+; *D42-Gal4*, *AP2σ^ang7/^AP2σ^KG02457^*: 2.5 ± 0.14, ns) failed to prevent the synaptic overgrowth in *σ2-adaptin* mutants (Extended Data Fig. 8-2). Consistent with this, expression of Rab11^WT^, Rab11^DN^, or Rab11^CA^ in *σ2-adaptin* mutant background did not rescue the functional defects in *σ2-adaptin* mutant (Extended Data Figs. 8-3, 8-4). Taken together, these results suggest that σ2-adaptin is crucial for the proper localization of Rab11 at the synapses and possibly required for the formation of the Rab11 positive recycling endosomes. We surmise that these recycling endosomes could be essential for targeting and distributing Tkv receptors at the synapses to regulate BMP signaling.

10.1523/ENEURO.0044-22.2022.f8-1 Extended Data Figure 8-1 Expressing a wild-type or constitutively active form of Rab11 does not alter the synaptic morphology. ***A–C***, Confocal images of NMJ synapses at muscles 6/7 NMJ at A2 hemisegment showing the synaptic growth in control (***A***), *D42-Gal4*-driven *UAS-Rab11-GFP* (***B***), and *D42-Gal4-*driven *UAS-Rab11^Q70L^-GFP* (***C***) double immunolabeled with a presynaptic vesicle marker, CSP (magenta), and a neuronal membrane marker, HRP (green), to mark the bouton outline. Expressing the wild-type and active form of Rab11 does not show morphological changes at the synapse. Scale bar in ***C*** represents 20 μm. ***D***, Histogram showing the average bouton number normalized to the muscle area from muscle 6/7 NMJ at A2 hemisegment in control animals (1.56 ± 0.06), *UAS-Rab11-GFP/+*; *D42-Gal4*/+ (1.56 ± 0.07), and *UAS-Rab11^Q70L^-GFP*; *D42-Gal4*/+ (1.58 ± 0.09). Error bar represents SEM; the statistical analysis was done using one-way ANOVA followed by *post hoc* Tukey’s test. ns, not significant. Download Figure 8-1, TIF file.

10.1523/ENEURO.0044-22.2022.f8-2 Extended Data Figure 8-2 Constitutively active Rab11 does not restore the NMJ morphological defects of *σ2-adaptin* mutants. ***A–D***, Confocal images of NMJ synapses at muscle 6/7 NMJ at A2 hemisegment showing synaptic growth in (***A***) Control animals, (***B***) *D42-Gal4 > Rab11^CA^*, (***C***) *AP2σ^KG02457^/AP2σ^ang7^*, and (***D***) *D42-Gal4 > Rab11^CA^, AP2σ^ang7^/AP2σ^KG02457^* double immunolabeled with a presynaptic synaptic vesicle marker, CSP (magenta) and a neuronal membrane marker, HRP (green) to mark the bouton outline. Scale bar in ***D*** represents 20 μm. ***E***, Histogram showing average bouton number normalized to the muscle area from muscle 6/7 NMJ at A2 hemisegment in control animals (1.05 ± 0.11), *D42-Gal4 > Rab11^CA^* (1.4 ± 0.10), *AP2σ^KG02457^/AP2σ^ang7^* (2.9 ± 0.14), and *D42-Gal4 > Rab11^CA^, AP2σ^ang7^/AP2σ^KG02457^* (2.5 ± 0.14). Error bar represents SEM; statistical analysis was done using one-way ANOVA followed by *post hoc* Tukey’s test. **p < 0.01; ns, not significant. Download Figure 8-2, TIF file.

10.1523/ENEURO.0044-22.2022.f8-3 Extended Data Figure 8-3 Overexpression of Rab11 does not restore the functional defects of *σ2-adaptin*. ***A***, Representative traces of mEJP in control, *D42 > Rab11^DN^*, *D42 > Rab11^WT^*, heteroallelic *AP2σ^KG02457^/AP2σ^ang7^*, *D42 > Rab11^DN^*, *AP2σ^KG02457^/AP2σ^ang7^*, and *D42 > Rab11^WT^*, *AP2σ^KG02457^/AP2σ^ang7^* larvae. ***B***, Representative tracesof EJP in control, *D42 > Rab11^DN^*, *D42 > Rab11^WT^*, heteroallelic *AP2σ^KG02457^/AP2σ^ang7^*, *D42 > Rab11^DN^*, *AP2σ^KG02457^/AP2σ^ang7^*, and *D42 > Rab11^WT^*, *AP2σ^KG02457^/AP2σ^ang7^* larvae. ***C***, Quantification of average EJP amplitude in control (44.42 ± 1.93), *D42 > Rab11^DN^* (53.89 ± 2.35), *D42 > Rab11^WT^* (52.71 ± 2.42), heteroallelic AP2σ^KG02457^/AP2σ^ang7^ (43.57 ± 4.52), *D42 > Rab11^DN^*, *AP2σ^KG02457^/AP2σ^ang7^* (38.03 ± 3.60), and *D42 > Rab11^WT^*, *AP2σ^KG02457^/AP2σ^ang7^* (32.92 ± 2.14). Error bars represent SEM; statistical analysis is based on one-way ANOVA followed by *post hoc* Tukey’s multiple-comparison test. **p < 0.01; ns, not significant. ***D***, Quantification of average mEJP amplitude in control (0.56 ± 0.01), *D42 > Rab11^DN^* (0.96 ± 0.08), *D42 > Rab11^WT^* (0.78 ± 0.05), heteroallelic *AP2σ^KG02457^/AP2σ^ang7^* (1.19 ± 0.05), *D42 > Rab11^DN^*, *AP2σ^KG02457^/AP2σ^ang7^* (0.13 ± 0.14), and *D42 > Rab11^WT^*, *AP2σ^KG02457^/AP2σ^ang7^* (1.46 ± 0.13). Error bars represent SEM; statistical analysis is based on one-way ANOVA followed by *post hoc* Tukey’s multiple-comparison test. **p < 0.01; ns, not significant. ***E***, Histograms showing average mEJP frequency in control (1.38 ± 0.10), *D42 > Rab11^DN^* (4.17 ± 0.47), *D42 > Rab11^WT^* (3.78 ± 0.31), heteroallelic *AP2σ^KG02457^/AP2σ^ang7^* (5.01 ± 0.23), *D42 > Rab11^DN^*, *AP2σ^KG02457^/AP2σ^ang7^* (4.33 ± 0.23), and *D42 > Rab11^WT^*, *AP2σ^KG02457^/AP2σ^ang7^* (4.06 ± 0.34). Error bars represent SEM; statistical analysis is based on one-way ANOVA followed by *post hoc* Tukey’s multiple-comparison test. **p < 0.01; ns, not significant. ***F***, Quantification of quantal content in control (50.56 ± 5.82), *D42 > Rab11^DN^* (53.14 ± 4.91), *D42 > Rab11^WT^* (66.11 ± 5.48), heteroallelic *AP2σ^KG02457^/AP2σ^ang7^* (38.95 ± 3.65), *D42 > Rab11^DN^*, *AP2σ^KG02457^/AP2σ^ang7^* (30.14 ± 3.03), and *D42 > Rab11^WT^*, *AP2σ^KG02457^/AP2σ^ang7^* (25.07 ± 0.93). Error bars represent SEM; statistical analysis is based on one-way ANOVA followed by *post hoc* Tukey’s multiple-comparison test. **p < 0.01; ns, not significant. Download Figure 8-3, TIF file.

10.1523/ENEURO.0044-22.2022.f8-4 Extended Data Figure 8-4 Constitutively active Rab11 does not restore the physiological defects of *σ2-adaptin* mutants. ***A***, Representative traces of mEJP in control, *D42-Gal4 > Rab11^CA^*, heteroallelic *AP2σ^KG02457^/AP2σ^ang7^*, and *D42-Gal4 > Rab11^CA^, AP2σ^ang7^/AP2σ^KG02457^* larvae. ***B***, Representative traces of EJP in control (*w^1118^*), *D42-Gal4 > Rab11^CA^*, heteroallelic *AP2σ^KG02457^/AP2σ^ang7^*, and *D42-Gal4 > Rab11^CA^, AP2σ^ang7^/AP2σ^KG02457^* larvae. ***C***, Quantification of average mEJP amplitude (mV) in control (0.67 ± 0.04), *D42-Gal4 > Rab11^CA^* (1.13 ± 0.17), heteroallelic *AP2σ^KG02457^/AP2σ^ang7^* (1.05 ± 0.09) and *D42-Gal4 > Rab11^CA^, AP2σ^ang7^/AP2σ^KG02457^* (1.30 ± 0.13). Error bars represent SEM; statistical analysis is based on one-way ANOVA followed by *post hoc* Tukey’s multiple-comparison test. **p < 0.01; ns, not significant. ***D***, Quantification of average EJP amplitude in control (44.52 ± 1.07), *D42-Gal4 > Rab11^CA^* (56.85 ± 4.221), heteroallelic *AP2σ^KG02457^/AP2σ^ang7^* (35.40 ± 1.925) and *D42-Gal4 > Rab11^CA^, AP2σ^ang7^/AP2σ^KG02457^* (41.41 ± 1.340). The error bars represent the SEM; statistical analysis is based on one-way ANOVA followed by *post hoc* Tukey’s multiple-comparison test. **p < 0.01; ns, not significant. ***E***, Histograms showing quantal content in the control (63.43 ± 4.81), *D42-Gal4 > Rab11^CA^* (26.88 ± 4.42), heteroallelic *AP2σ^KG02457^/AP2σ^ang7^* (47.08 ± 4.13) and *D42-Gal4 > Rab11^CA^, AP2σ^ang7^/AP2σ^KG02457^* (37.38 ± 5.83). The error bars represent the SEM; statistical analysis is based on one-way ANOVA followed by *post hoc* Tukey’s multiple comparison test. **p < 0.01; ns, not significant. Download Figure 8-4, TIF file.

In conclusion, *σ2-adaptin* and *Rab11* increase Tkv accumulation and BMP signaling to generate similar NMJ phenotypes via independent pathways.

## Discussion

Compromised endocytosis not only perturbs synaptic transmission but also has been implicated in deregulating synaptic growth as demonstrated in *Endo*, *Synj, nwk*, *shi*, *Clc*, *brat*, and *σ2-adaptin* mutant NMJs (Rikhy et al., 2002; Verstreken et al., 2002; Verstreken et al., 2003; Choudhury et al., 2016). The underlying molecular mechanism by which these proteins regulate synaptic growth, however, has only been demonstrated for *nwk* and *brat* (O’Connor-Giles et al., 2008; Shi et al., 2013). Our previous study on *σ2-adaptin* mutants showed no change in levels of endocytic proteins like Endo, Synj, and Dyn (Choudhury et al., 2016), prompting us to investigate the role of σ2-adaptin in synaptic growth signaling. Mutations that affect endocytosis, in general, show synaptic overgrowth and increased BMP effector, pMad. The signaling output of growth-regulating pathwaysis often dependent on intracellular traffic that in part is dependent on endocytosis of activated receptors, ultimately impinging on the BMP, JNK, or Wingless pathways (Shi et al., 2013; Deshpande and Rodal, 2016). Here, we show for the first time that σ2-adaptin genetically interacts with BMP signaling pathway at the synapse. Loss of σ2-adaptin leads to accumulation of Tkv receptor at the NMJ. Additionally, we provide evidence that σ2-adaptin regulates the localization of recycling endosomal protein Rab11.

### σ2-adaptin genetically interacts with BMP pathway to regulate neuronal BMP signaling

Endosomal trafficking of BMP receptors is a crucial regulatory mechanism that controls synaptic growth (Rodal et al., 2011). Various proteins interact with BMP receptors to facilitate or attenuate the BMP-dependent signaling cascade (McCabe et al., 2004; XW Zhang et al., 2017). Endocytic proteins appear to be fascinating candidates as BMP receptor interactors. *Drosophila* loss-of-function endocytic mutants correlate with elevated BMP signaling and neuronal overgrowth phenotype (Dickman et al., 2006; Rodal et al., 2011; Deshpande and Rodal, 2016). Consistent with this, we show that increased Tkv levels at the NMJ result in elevated BMP signaling in *σ2-adaptin* mutants. If the BMP pathway is responsible for the synaptic overgrowth in *σ2-adaptin* mutants, we reasoned that reducing the levels of BMP signaling components should rescue the NMJ phenotype. In agreement with this, we found that partially reducing BMP receptors Tkv, Wit, and cytosolic co-Smad molecule, Medea, significantly rescues the NMJ defects in *σ2-adaptin* mutants. Our data reveal that σ2-adaptin genetically interacts with the negative regulator of BMP signaling, the inhibitory Smad, Dad. Transheterozygotes of *Dad* and *σ2-adaptin* mutants have increased number of boutons compared with heterozygotes of either mutant alone. Consistent with this inference, neuronal expression of *UAS-Dad* in *σ2-adaptin* mutant background significantly reduces the synaptic overgrowth phenotype. However, reducing BMP signaling only partially reduces the bouton size in *σ2-adaptin* mutants. There could be at least two plausible explanations for the partial rescue of the bouton size: first, removing only one copy of *tkv* may not be sufficient for rescuing this defect. Since mutating both copies of *tkv* results in embryonic lethality, we could not test the epistatic interactions by removing both the copies of Tkv in *σ2-adaptin* mutants. Second, σ2-adaptin may regulate NMJ bouton size through a different signaling pathway, which remains to be elucidated. Overall, our data suggest that σ2-adaptin negatively regulates the BMP growth signaling pathway to attenuate synaptic growth.

### σ2-adaptin regulates trafficking of Thickveins at the NMJ

BMP signaling has been extensively studied in the context of neuronal growth in which the activated Tkv is endocytosed and fuse with the early endosomes, where it activates downstream signaling molecules. The signaling is attenuated when these activated receptor-containing vesicles recycle back to the plasma membrane or fuse with lysosomes for degradation (Rodal et al., 2011; Smith et al., 2012). Trafficking of these receptors into and out of such endosomes provides an additional tier for spatial and temporal modulation of signal transduction. The members of the Rab family of small GTPases regulate various stages of endocytosis (Kelly et al., 2012). Our immunocytochemistry data show elevated Tkv receptor levels at the synapses and motor neuron soma (data not shown) of *σ2-adaptin* mutants. Besides, levels of Rab11 (known for its role in the recycling of Tkv receptor) are reduced by half in *σ2-adaptin* mutant synapses.

Interestingly, levels of early and late endosomes marked with Rab5 and Rab7, respectively, remain unaffected at the *σ2-adaptin* mutant synapses. The intensity profile of Tkv and HRP across the bouton shows that *σ2-adaptin* mutant has a higher intensity of Tkv at the membrane, indicating that a significant proportion of the Tkv receptors are accumulated at the presynaptic membrane. The pattern of Tkv enrichment in *σ2-adaptin* and *Rab11* mutants were distinct. While *σ2-adaptin* mutant synapses showed Tkv enriched at synaptic membranes, *Rab11* mutants had a rather punctate distribution within the bouton. The differential distribution of these proteins could be because of their distinct roles in the neurons. While σ2-adaptin plays a critical role in retrieving the SV-membrane from the presynaptic membrane, Rab11 is a component of the endolysosomal machinery. A clear separation of Tkv signals/pixels distinguishing plasma membrane Tkv from endosomal Tkv is challenging at the NMJ, given the resolution limit of the confocal system. The difference in Tkv distribution in *σ2-adaptin* and *Rab11* mutants also argues for additional pathways, other than Rab11, through which σ2-adaptin may regulate BMP signaling.

Tkv receptors in *σ2-adaptin* mutants could be accumulated either at the plasma membrane because of inefficient CME or at the early endosomes caused by inefficient recycling. If Tkv were accumulated at the early endosomes, we would expect greater colocalization with Rab5, which is not the case. However, based on reduced Rab11 staining at the *σ2-adaptin* mutant synapses, we conclude that the portion of the receptors in Rab5 positive early endosomes fail to recycle back to the plasma membrane. This conclusion also fits with previous observations that defective CME results in the accumulation of endosome-like structures in cultured hippocampal neurons (Kononenko et al., 2014) and is substantiated by our electron microscopy data. The link between clathrin-mediated dynamin-dependent endocytosis and BMP signaling is still a contentious topic. A recent study using human umbilical vein endothelial cells (HUVECs) has shown that treating these cells with BMP-9 triggered Caveolin-1 and dynamin-2-mediated endocytosis of its receptor, activin-like kinase 1 (ALK-1). Surprisingly, this ALK-1 endocytosis was not mediated by Clathrin heavy chain (Tao et al., 2020). At the *Drosophila* NMJ, endocytosis attenuates BMP signaling (O’Connor-Giles et al., 2008), whereas in *Drosophila* wing discs and intestinal stem cells, endocytosis facilitates the signaling cascade by internalizing Tkv (Gui et al., 2016; Tracy Cai et al., 2019), pointing toward a tissue-specific mechanism. Our results suggest a model where bulk membrane endocytosis is insufficient in removing Tkv from the plasma membrane; besides, the synapses in *σ2-adaptin* mutants fail to recycle remaining receptors from early endosomes leading to enhanced signaling and NMJ growth defects.

### Functional and morphologic aspects of σ2-adaptin-mediated BMP signaling can be discriminated

Morphologic features of synapses often dictate functional outcomes, and physiological analyses of BMP signaling mutants reveal the same. In *wit* mutants, the size of the NMJ is significantly reduced with concomitant reduced evoked excitatory potentials (Aberle et al., 2002; Marqués et al., 2002). Analyses of *tkv* and *sax*, co-Smad *Medea*, and transcription factor *Mad* showed that these NMJs have smaller synapses with severe functional deficits (McCabe et al., 2004). Consistent with the role of BMP-signaling in regulating NMJ growth, *gbb* mutant larvae also exhibit shorter NMJs with severely reduced evoked potentials (McCabe et al., 2003). *σ2-adaptin* mutant synapses show a modest reduction in evoked potentials, and the protein is dispensable for maintaining basal synaptic transmission (Choudhury et al., 2016). However, a rundown of EJP amplitudes during high-frequency stimulation in synaptic mutants implicated in CME, such as *Endo*, *Synj*, and *Dap160*, show a rapid stimulus-dependent decline in EJP amplitude that recovers following a period of rest after the high-frequency stimulation paradigm (Verstreken et al., 2003; Koh et al., 2004). In our previous study, we reported that *σ2-adaptin* mutants do not recover from synaptic depression even after the 90s rest after the cessation of high-frequency stimulation (Choudhury et al., 2016). This observation suggested that in addition to its requirement in synaptic membrane retrieval, the σ2-adaptin function is also required during the much slower process of SV trafficking, possibly at one of the rate-limiting steps in SV regeneration. This conclusion is supported by our EM data that shows the accumulation of endosome-like structures more frequently at the mutant synapses when compared with *w^1118^* controls. EJP and high-frequency recordings from *σ2-adaptin* mutant synapses with one copy of *tkv^7^* did not show any rescue in synaptic function. These data drive the conclusion that partial reduction of BMP pathway components can only rescue morphologic defects in *σ2-adaptin* mutants but not functional aspects and that morphologic and functional deficits can be discriminated in these mutants. Besides, the partial rescue of bouton size and bouton clustering at the NMJ argues for possible deregulation of multiple signaling pathways in *σ2-adaptin* mutants that remain to be explored.

Our study uncovers and extends the existing knowledge of synaptic growth signaling and endocytosis. We provide four lines of evidence on the critical role of σ2-adaptin in modulating BMP-dependent synaptic growth signaling at the *Drosophila* NMJ. First, we show using genetics that the morphologic defects in *σ2-adaptin* mutant synapses can be partially rescued by introducing a mutant copy of the BMP receptors, *tkv*, and *wit*. We also show a direct epistatic interaction between σ2-adaptin and the inhibitory Smad, Dad. Second, using immunohistochemistry, we show that *σ2-adaptin* mutant synapses accumulate Tkv at the plasma membrane and some of these receptors that are endocytosed and make it to the early endosomes fail to recycle back to the plasma membrane. Third, our electrophysiology data establish that morphologic and functional defects can be discriminated in *σ2-adaptin* mutants. Finally, our electron micrographs provide evidence for the presence of large endosomes and support our conclusion that σ2-adaptin is critically required at a later step of vesicle regeneration following endocytosis from the plasma membrane.

While this study does not report a direct biochemical or epistatic interaction between σ2-adaptin and Rab11, we observed a significant reduction in Rab11 immunoreactivity in *σ2-adaptin* mutant synapses that could be restored by neuronal expression of *σ2-adaptin* transgenes. However, the structural and functional defects of *σ2-adaptin* mutants could not be restored by neuronal expression of Rab11^WT^ or Rab11^CA^. This rules out the notion that σ2-adaptin phenotypes result from the observed reduction of Rab11. The *Rab11* mutant used in this study is a hypomorph with substantial Rab11 protein being detected by us and others (Khodosh et al., 2006). This precludes further epistatic analyses of this mutant. The fact that Rab11 protein could still be detected in these *Rab11* hypomorphic mutants may explain why pMad levels were not as upregulated when compared with *σ2-adaptin* mutants. Finally, the separation of Tkv immunoreactivity from the synaptic membrane versus that of the endosomal membrane is challenging, given the resolution of the imaging system used here. Our electron micrographs, however, convincingly show an accumulation of endosome-like structures close to the plasma membrane in *σ2-adaptin* mutant boutons. The endosome-like structures observed in the σ2-adaptin mutants are strikingly similar to structures previously reported for clathrin (Khodosh et al., 2006), AP180 (B Zhang et al., 1998), and Rab11 (Inoshita et al., 2017), among others. Thus, Tkv receptors could likely be enriched in these endosome-like compartments.

**Figure 9.**
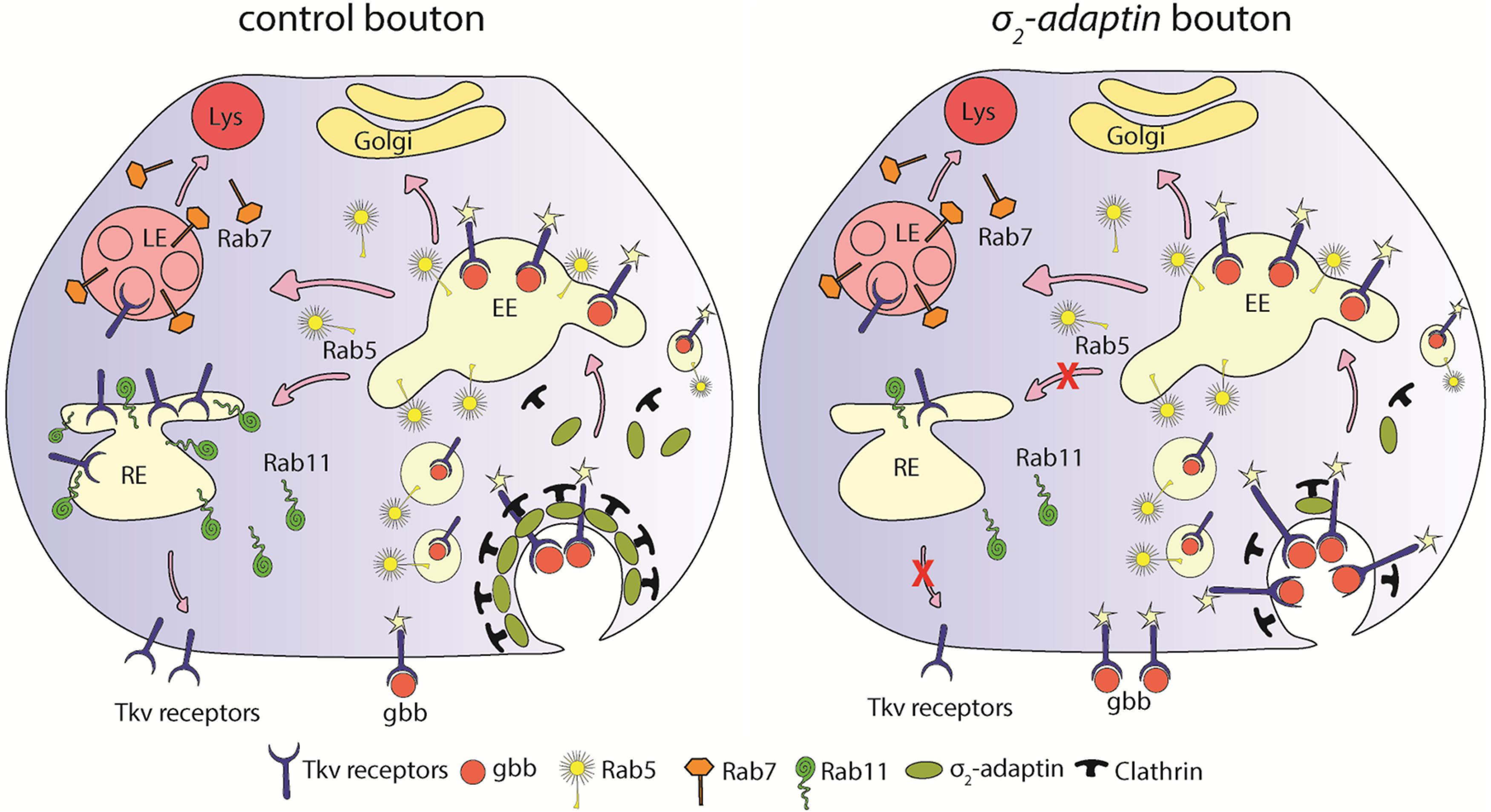
Model depicting the role of σ2-adaptin in BMP receptor trafficking at the NMJ. The model depicts a novel function of σ2-adaptin/AP2 in Tkv receptor trafficking at the *Drosophila* NMJ. Retrograde NMJ growth signaling in *Drosophila* involves Gbb ligand that is secreted from postsynaptic muscles and binds to BMP receptors on the presynaptic membrane to activate them (Aberle et al., 2002; McCabe et al., 2003). Activated receptors are then internalized through CME and fuse with early endosomes to trigger the downstream signaling cascade (Hartung et al., 2006). From early endosomes, receptors either get sorted to the Rab11-positive recycling endosomes that recycle them back to the presynaptic membrane or are sorted for lysosomal degradation (Liu et al., 2014; Deshpande and Rodal, 2016). Depletion of σ2-adaptin/AP2-complex leads to enrichment of Tkv receptors at the presynaptic membrane and/or in the early endosomes leading to elevated BMP signaling resulting in synaptic overgrowth. It is also likely that reduced synaptic Rab11 levels in σ2-adaptin might perturb Rab11-mediated recycling of Tkv leading to enhanced BMP-signaling.

We propose a model in which σ2-adaptin/AP2-complex is required for the attenuation of BMP signaling at the *Drosophila* NMJ. In the absence of AP2, recycling of Tkv is compromised, which results in its enrichment at the presynaptic membrane and/or in the early endosomes leading to elevated BMP signaling and synaptic overgrowth (Fig. 9). This study thus opens new avenues where the role of other CME components and their interaction with various growth signaling pathways can be studied. Since receptor localization and regulation appear to be the central theme in modulating BMP signaling and synapse growth, it will be interesting to perform structure-function analyses of BMP receptors and identify key residues/motifs that interact with AP2 and facilitate its endocytosis. Mutating tyrosine-based signal (YXXϕ) and dileucine-based signal ([DE]XXXL[LI]) motifs in Tkv and Wit could lead to further understanding of these intricate interactions.

## Synthesis

Reviewing Editor: Matthew Grubb, King’s College London Decisions are customarily a result of the Reviewing Editor and the peer reviewers coming together and discussing their recommendations until a consensus is reached. When revisions are invited, a fact-based synthesis statement explaining their decision and outlining what is needed to prepare a revision will be listed below. The following reviewer(s) agreed to reveal their identity: Juan Jose Perez-Moreno. Note: If this manuscript was transferred from JNeurosci and a decision was made to accept the manuscript without peer review, a brief statement to this effect will instead be what is listed below.

Reviewer 1:

The manuscript details phenotypes arising from loss of σ2-adaptin at the Drosophila larval NMJ. Primarily, they provide novel insight into its role of regulating Tkv trafficking so as to attenuate excessive BMP signaling. A degree of overlap with Rab11 phenotypes lead the authors to link this the role of σ2-adaptin to Rab11 localization and activation. However, attempts to restore Rab11 at the NMJ fail to rescue σ2-adaptin loss of function phenotypes

The manuscript requires considerable editing prior to publication. The observations regarding σ2-adaptin phenotypes are of sufficient interest for publication, but the following needs to be resolved.

1) The manuscript is rather poorly written, with many grammatical errors throughout; albeit without hindering comprehension of the manuscript.

2) There is persistent over-interpretation of links between σ2-adaptin and Rab11. In the rebuttal to the previous review, the authors state: “While AP2σ might regulate Rab11 activity; however, this is not a conclusion of this manuscript"

However, this exact point does remain a central conclusion of the current manuscript. Indeed, the abstract states “Expression of Rab11 does not restore the synaptic defects of σ2-adaptin mutations suggesting that σ2-adaptin is essential for regulating Rab11 activity. Thus, we propose a model in which AP2 regulates Tkv internalization and endosomal recycling through a process that requires Rab11 activity to control synaptic growth.” This appears to directly contradict the claim in rebuttal.

The statement in the rebuttal “Overexpressing a wild type or a constitutively active Rab11 transgene in neurons in an AP2-σ mutant background most likely overwhelms the system rendering Rab11 inactive.” This assertion needs to be explained. It is not clear why this is the case, and upon what evidence does this assertion stand?

Moreover, the following concluding statement in the introduction is not supported by the evidence. “Finally, we show that synaptic enrichment of Tkv receptors in the σ2-adaptin mutants is a result of defective Rab11-dependent vesicular trafficking”. Again, the σ2-adaptin mutant phenotype has not been experimentally verified as arising because of the coincident loss of Rab11; the Rab11 restoration in σ2-adaptin mutants did not rescue the phenotype.

Finally, as stated by the author, “While σ2-adaptin mutants showed Tkv enrichment at the synaptic membranes, Rab11 mutants had punctate Tkv accumulation within the boutons, and does not show enrichment at the presynaptic membrane (Figure S2).” The authors do not clarify why these phenotypes would be distinct, if σ2-adaptin is acting through Rab11 activation to modulate Tkv trafficking.

This needs to be addressed in a revised manuscript that better clarifies what the data does support, the limitations of any experimental approaches to resolve the problem, possible work-arounds to actually resolve any interactions between Rab11 and σ2-adaptin, and discriminate this from what the authors offer as conjecture as to the proposed mechanisms.

Minor points

1) The sentence on lines 62-65 requires at least one citation.

2) The sentence on lines 66-67 is not accurate; Gbb is also secreted from motor neurons.

3) The sentence on lines 72-74 is not entirely accurate, as the role of signaling endosomes is not invoked in reference to retrograde signaling. This is curious, because the manuscript focuses on mechanisms of endocytosis.

4) The introduction states: “Analysis of vesicular trafficking using endosomal markers shows that σ2-adaptin regulates the localization of Rab11 at the NMJ synapse.” However, there is a loss of Rab11 at the NMJ in σ2-adaptin mutants, rather than evidence for mislocalization. This is a subtle point but it is not clear why Rab11 is not present, with mislocalization being only one explanation.

5) The sentence “Western blot analysis revealed that total levels of Rab11 protein in σ2-adaptin mutants were not altered (Figure S5).” Altered compared to what? This needs clarity.

Reviewer 2:

It is well-known the existence of a link between endocytosis and BMP signaling pathway, as well as its importance in synaptic growth and function. Here, authors provide a novel role for the clathrin-mediated endocytosis (CME) (in particular for AP2-α) in regulating BMP signaling. The key observation is that AP2-σ mutants show impaired Rab11-mediated recycling of Tkv receptors, leading to enhanced BMP signaling. This is a step forward in the study of the presynaptic regulation, and it should be of interest for the scientific community in this field.

In general, the current version of the manuscript results well detailed and presents well-supported conclusions. However, I have a few concerns that should be addressed:

Major comments:

1. In Fig. 7M it is shown that although Rab11 KO or OE does not produces a significant increase of pMad levels, it does not phenocopy the pMad levels observed in AP2σ mutants. This should be discussed, since it suggests that Rab11-positive recycling endosomes is not the only pathway by which AP2σ regulates Tkv levels.

On the other hand, a key related question is whether Rab11[ex2/93Bi] is a null mutant allele. According authors data, Rab11[ex2/93Bi] animals still show some Rab11 levels. This was observed by immunofluorescence (Fig. 6S) and by western-blot (Fig. S5), and it might explain why Rab11[ex2/93Bi] animals do not increase pMad levels as much as AP2σ mutants. This should be properly discussed and highlighted by the authors.

2. They key message from the article is the σ2-adaptin/AP2-complex requirement for the formation of Rab11 positive recycling endosomes. However, Authors show that expression of the WT form of Rab11 cannot rescue synaptic overgrowth in AP2σ mutants (Fig. 8E-F) or synaptic function (Fig. S4). The proposed model (Fig. 9) might imply that AP2σ not only regulate Rab11 levels (Fig. 6S), but also its activity/localization. Therefore, it would be critical to study whether constitutively active form of Rab11 (already used in Fig. S3) can rescue synaptic overgrowth and mEJP defects in AP2σ mutants.

Minor comments:

It results quite challenging for the reader the way authors present the different used alleles. The first example of this is found from line 153 to 159, where a group of allele combinations are presented, but it is not provided a proper description of them. For example, are AP2σ[KG02457] and AP2σ[ang7] independently-generated null mutant alleles for AP2σ gene? Also, references supporting the loss of function should be cited for each allele used in the article.

There is no data supporting a defect between endocytosis and formation of early endosomes in AP2σ mutants. However, this is shown in Fig. 9 model (Visual abstract). Therefore, authors should leave this step as unaffected in the Figure, and only conclude what it is supported by their data: that early endosomes fail to recycle back to the plasma membrane in AP2σ mutants.

## Author Response

We would like to thank the reviewers for their interest in our manuscript and providing us with a very detailed and valuable suggestions that have helped us improve this manuscript. We have addressed the two main issues pointed by the editor:

a) The Rab11 hypomorphic combination phenocopies the σ2-adaptin mutants (Figure 8). Hence recycling endosomes could be playing role in the regulation of BMP-signalling. We have mentioned this in the revised manuscript. However, we agree that the underlying mechanisms needs to be explored further.

b) We have performed additional experiments related to rescue with Rab11CA in response to yours as well as Reviewer 2 suggestions. We found that expression of Rab11CA does not rescue the morphological or the functional defects of AP2σ mutants. The relevant data has been incorporated as Figure 8-2 and 8-4.

c) We have removed our claim that AP2 functions via Rab11 in the revised manuscript. We have changed the title of the manuscript to incorporate this aspect.

Please find below our detailed point-by-point responses to their comments:

1. Manuscript formatting for eNeuro

1.1 Significance Statement is missing from the manuscript text. Please add it to the text.

Response: The significance statement has now been added.

1.2 Please re-organize the manuscript so that the Materials and Methods follows directly after the Introduction.

Response: The Materials and Methods section has been reorganized and follows directly after the Introduction.

2. Editor’s comments

2.1 The data show that Rab11-positive recycling endosomes is not the only pathway by which AP2σ regulates BMP signaling pathway, since Rab11 KO do not phenocopy AP2σ KO.

Response: We agree with the editor that Rab11-positive recycling endosomes may not be the only pathway by which AP2σ regulates BMP signaling pathway. In terms of the NMJ phenotype, we do observe that Rab11 KO animals phenocopy AP2σ mutants. This is documented in Figure 8 and the quantifications of bouton number/ muscle area and average bouton area (Figure 8E and 8F respectively) demonstrate this. The synaptic pMAD levels are also high for both Rab11 KO animals and AP2σ mutants as shown in Figure 7M. The title of the manuscript has also been changed to “AP2 regulates Thickveins trafficking to attenuate NMJ growth signaling in Drosophila” to avoid any over-interpretation of the link between σ2-adaptin and Rab11.

2.2 To claim that AP2σ regulates NMJ growth and function through Rab11, the reported defects should be rescued by expressing the constitutively active form of Rab11. This construct has already been used in the paper (Fig. S3) in a WT background. Results from this rescue experiment should be reported, regardless of the result obtained.

Response: We thank the editor for suggesting this experiment and the results for the same are now included in the results under sub-section “Rab11 mutants phenocopy BMP-signaling and NMJ defects of σ2-adaptin”. The data pertaining to this experiment has been incorporated as Figure 8-2 and 8-4.

3. Reviewer 1 comments

The manuscript details phenotypes arising from loss of σ2-adaptin at the Drosophila larval NMJ. Primarily, they provide novel insight into its role of regulating Tkv trafficking so as to attenuate excessive BMP signaling. A degree of overlap with Rab11 phenotypes lead the authors to link this role of σ2-adaptin to Rab11 localization and activation. However, attempts to restore Rab11 at the NMJ fail to rescue σ2-adaptin loss of function phenotypes

The manuscript requires considerable editing prior to publication. The observations regarding σ2-adaptin phenotypes are of sufficient interest for publication, but the following needs to be resolved.

3.1 The manuscript is rather poorly written, with many grammatical errors throughout; albeit without hindering comprehension of the manuscript.

Response: We thank the reviewer for appreciating the novelty in our manuscript. We also thank the reviewer for pointing out the grammatical errors and typos in the manuscript. The revised version of the manuscript has been considerably edited and corrected for grammatical errors to the best of our abilities. We hope that the revised version will be easier to comprehend and appreciated by the reviewer.

3.2 There is persistent over-interpretation of links between σ2-adaptin and Rab11. In the rebuttal to the previous review, the authors state: “While AP2σ might regulate Rab11 activity; however, this is not a conclusion of this manuscript.”

However, this exact point does remain a central conclusion of the current manuscript. Indeed, the abstract states “Expression of Rab11 does not restore the synaptic defects of σ2-adaptin mutations suggesting that σ2-adaptin is essential for regulating Rab11 activity. Thus, we propose a model in which AP2 regulates Tkv internalization and endosomal recycling through a process that requires Rab11 activity to control synaptic growth.” This appears to directly contradicting the claim in rebuttal. The statement in the rebuttal “Overexpressing a wild type or a constitutively active Rab11 transgene in neurons in an AP2-σ mutant background most likely overwhelms the system rendering Rab11 inactive.” This assertion needs to be explained. It is not clear why this is the case, and upon what evidence does this assertion stand?

Response: We agree with the reviewer that there was a persistent over-interpretation of the link between σ2-adaptin and Rab11 in our manuscript. We have ensured in the revised manuscript that no such over-interpretation is made and have corrected the text in the abstract, results and elsewhere in the manuscript. As it stands now, we report the decrease in synaptic Rab11 immunoreactivity in AP2σ mutants as an observation without claiming that σ2-adaptin is essential for regulating Rab11 activity. We have also revised the title of our manuscript to “AP2 regulates Thickveins trafficking to attenuate NMJ growth signaling in Drosophila” to avoid any over-interpretation of the link between σ2-adaptin and Rab11.

In response to the assertion “Overexpressing a wild type or a constitutively active Rab11 transgene in neurons in an AP2-σ mutant background most likely overwhelms the system rendering Rab11 inactive", we would like to clarify that this assertion is based on the fact that the synapse is a crowded cellular milieu and some of these synaptic proteins are lowly expressed. In general, over-expressing any transgene in neurons in a wild-type background or otherwise can result in cellular stress and might interfere with protein-protein interactions at the synapse. We do agree that the assertion “Rab11 overexpression in neurons most likely overwhelms the system rendering Rab11 inactive” could be inaccurate. We have removed this sentence in the revised manuscript.

3.3 Moreover, the following concluding statement in the introduction is not supported by the evidence. “Finally, we show that synaptic enrichment of Tkv receptors in the σ2-adaptin mutants is a result of defective Rab11-dependent vesicular trafficking”. Again, the σ2-adaptin mutant phenotype has not been experimentally verified as arising because of the coincident loss of Rab11; the Rab11 restoration in σ2-adaptin mutants did not rescue the phenotype.

Response: We thank the reviewer for pointing this out and agree that our data does not support the conclusion that synaptic enrichment of Tkv receptors in the σ2-adaptin mutants is a result of defective Rab11-dependent vesicular trafficking. It is also true that Rab11 restoration in σ2-adaptin mutants did not rescue the phenotype. We have now removed this concluding statement from the introduction.

3.4 Finally, as stated by the author, “While σ2-adaptin mutants showed Tkv enrichment at the synaptic membranes, Rab11 mutants had punctate Tkv accumulation within the boutons, and does not show enrichment at the presynaptic membrane (Figure S2).” The authors do not clarify why these phenotypes would be distinct, if σ2-adaptin is acting through Rab11 activation to modulate Tkv trafficking.

This needs to be addressed in a revised manuscript that better clarifies what the data does support, the limitations of any experimental approaches to resolve the problem, possible work-arounds to actually resolve any interactions between Rab11 and σ2-adaptin, and discriminate this from what the authors offer as conjecture as to the proposed mechanisms.

Response: We thank the reviewer for bringing this to our notice. Indeed, the Tkv immunoreactivity pattern in σ2-adaptin and Rab11 mutants are different. We have discussed these distinct phenotypes in our revised manuscript in the discussion under sub-section “σ2-adaptin regulates trafficking of Thickveins at the NMJ”.

As suggested by the reviewer, we have also added a short paragraph in the section that address the limitations of our experimental approaches.

3.5 Minor points

3.5.1 The sentence on lines 62-65 requires at least one citation.

Response: We have now added three citations (Keshishian; Trends Neurosci. 2004, O’Connor-Giles; Neuron 2008 and Sulkowski et. al.; PLoS Genet. 2016) for the sentence on lines 62-65 in the revised manuscript.

3.5.2 The sentence on lines 66-67 is not accurate; Gbb is also secreted from motor neurons.

Response: We have now corrected the sentence on lines 66-67 in the revised manuscript to “At the Drosophila NMJ, the retrograde BMP signaling is initiated by secretion of glass bottom boat (Gbb) from the muscle and the motor neurons” in the introduction and provided appropriate references.

3.5.3 The sentence on lines 72-74 is not entirely accurate, as the role of signaling endosomes is not invoked in reference to retrograde signaling. This is curious, because the manuscript focuses on mechanisms of endocytosis.

Response: We have now replaced the sentence on lines 72-74 in the revised manuscript to “Following BMP activation, these receptors are endocytosed for retrograde transport to the motor neuron nuclei in the soma” in the introduction and added appropriate references.

3.5.4 The introduction states: “Analysis of vesicular trafficking using endosomal markers shows that σ2-adaptin regulates the localization of Rab11 at the NMJ synapse.” However, there is a loss of Rab11 at the NMJ in σ2-adaptin mutants, rather than evidence for mislocalization. This is a subtle point but it is not clear why Rab11 is not present, with mislocalization being only one explanation.

Response: We have now modified this sentence in the introduction to “Analysis of vesicular trafficking using endosomal markers show that Rab11 is reduced in σ2-adaptin mutant NMJ synapses.” This is further discussed in the results section.

3.5.5 The sentence “Western blot analysis revealed that total levels of Rab11 protein in σ2-adaptin mutants were not altered (Figure S5).” Altered compared to what? This needs clarity.

Response: Western blot analysis revealed that total levels of Rab11 protein in σ2-adaptin mutants were not altered compared to wild type controls (Figure 6-1). This sentence is also corrected in the revised manuscript.

4. Reviewer 2 comments

It is well-known the existence of a link between endocytosis and BMP signaling pathway, as well as its importance in synaptic growth and function. Here, authors provide a novel role for the clathrin-mediated endocytosis (CME) (in particular for AP2-α) in regulating BMP signaling. The key observation is that AP2-σ mutants show impaired Rab11-mediated recycling of Tkv receptors, leading to enhanced BMP signaling. This is a step forward in the study of the presynaptic regulation, and it should be of interest for the scientific community in this field. In general, the current version of the manuscript results well detailed and presents well-supported conclusions. However, I have a few concerns that should be addressed:

Major comments:

4.1 In Fig. 7M it is shown that although Rab11 KO or OE does not produce a significant increase of pMad levels, it does not phenocopy the pMad levels observed in AP2σ mutants. This should be discussed, since it suggests that Rab11-positive recycling endosomes is not the only pathway by which AP2σ regulates Tkv levels.

Response: We thank the reviewer for appreciating the novel role of clathrin-mediated endocytosis (CME) (in particular, for AP2σ) in regulating BMP signaling in our submitted manuscript. We also thank the reviewer for considering this a step forward in the study of the presynaptic regulation, and to be of interest for the scientific community in this field. We do agree that Rab11-positive recycling endosomes is not the only pathway by which AP2σ regulates Tkv levels. This has now been extensively discussed under sub-section “σ2-adaptin regulates trafficking of Thickveins at the NMJ” of discussion.

4.2 On the other hand, a key related question is whether Rab11[ex2/93Bi] is a null mutant allele. According authors data, Rab11[ex2/93Bi] animals still show some Rab11 levels. This was observed by immunofluorescence (Fig. 6S) and by western-blot (Fig. S5), and it might explain why Rab11[ex2/93Bi] animals do not increase pMad levels as much as AP2σ mutants. This should be properly discussed and highlighted by the authors.

Response: We thank the reviewer for pointing this out. Indeed, in the original paper (Khodosh et. al.; Development 2006), the authors show reduced levels of Rab11 protein meaning that Rab11[ex2/93Bi] is not a null allele. This is also reproducible in our hands as Rab11[ex2/93Bi] animals still show some Rab11, both by immunofluorescence and Western blot. This actually explains why Rab11[ex2/93Bi] animals do not show increased pMad levels as much as AP2σ mutants. This is now discussed under sub-section “Functional and morphological aspects of σ2-adaptin-mediated BMP signaling can be delineated” of discussion.

4.3 They key message from the article is the σ2-adaptin/AP2-complex requirement for the formation of Rab11 positive recycling endosomes. However, Authors show that expression of the WT form of Rab11 cannot rescue synaptic overgrowth in AP2σ mutants (Fig. 8E-F) or synaptic function (Fig. S4). The proposed model (Fig. 9) might imply that AP2σ not only regulate Rab11 levels (Fig. 6S), but also its activity/localization. Therefore, it would be critical to study whether constitutively active form of Rab11 (already used in Fig. S3) can rescue synaptic overgrowth and mEJP defects in AP2σ mutants.

Response: As suggested by the reviewer, we have now performed this experiment and the results for the same are now included in the results under sub-section “Rab11 mutants phenocopy BMP-signaling and NMJ defects of σ2-adaptin”. We observe that constitutively active form of Rab11 does not rescue the synaptic overgrowth and mEJP defects in AP2σ mutants (Figure 8-2 and 8-4).

4.4 Minor comments:

4.4.1 It results quite challenging for the reader the way authors present the different used alleles. The first example of this is found from line 153 to 159, where a group of allele combinations are presented, but it is not provided a proper description of them. For example, are AP2σ[KG02457] and AP2σ[ang7] independently-generated null mutant alleles for AP2σ gene? Also, references supporting the loss of function should be cited for each allele used in the article.

Response: We thank the reviewer for referring to lines 153 to 159 where different alleles of AP2σ are mentioned. We understand that this would pose a challenge to the readers unfamiliar with our previous work that discusses the characterization of these alleles (Choudhury et. al.; Genetics 2016). We have now added the reference for these alleles in the text where they appear (Results; sub-section “σ2-adaptin genetically interacts with regulators of BMP signaling”).

4.4.2 There is no data supporting a defect between endocytosis and formation of early endosomes in AP2σ mutants. However, this is shown in Fig. 9 model (Visual abstract). Therefore, authors should leave this step as unaffected in the Figure, and only conclude what it is supported by their data: that early endosomes fail to recycle back to the plasma membrane in AP2σ mutants.

Response: As suggested by the reviewer, we have now revised our model where we have left the step between endocytosis and formation of early endosomes in AP2σ mutants unaffected. The revised model shows only early endosomes fail to recycle back to the plasma membrane in AP2σ mutants.
