# Supplementary figures and images for: AP2 Regulates Thickveins Trafficking to Attenuate NMJ Growth Signaling in Drosophila
Source: eNeuro. 2022 Oct 11;9(5):ENEURO.0044-22.2022. doi: 10.1523/ENEURO.0044-22.2022 (PMC9581581; doi:10.1523/ENEURO.0044-22.2022)

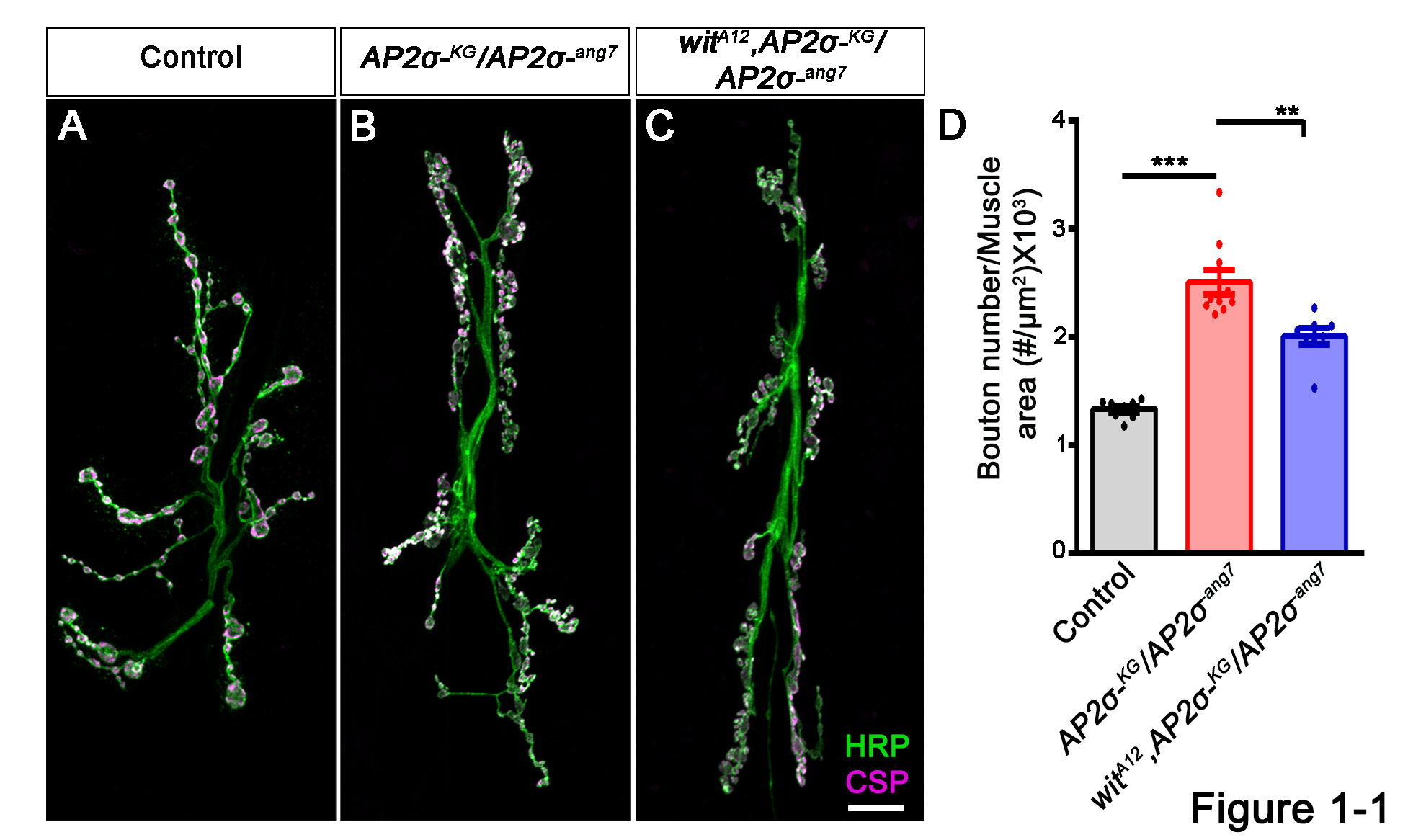

Supplement: Extended Data Figure 1-1 — Reducing levels of Wit receptor rescues the bouton number in σ2-adaptin mutant. A–C, Confocal images of NMJ synapses at muscles 6/7 NMJ at A2 hemisegment showing the synaptic growth in control (A), AP2σKG02457/AP2σang7 (B), witA12, AP2σKG02457/AP2σang7 (C), double immunolabeled with a presynaptic vesicle marker, CSP (magenta), and a neuronal membrane marker, HRP (green), to mark the bouton outline. Reducing the levels of BMP Type II receptor in the AP2σKG02457/AP2σang7 background reduces the synaptic overgrowth. Scale bar in C represents 10 μm. D, Histogram showing the average bouton number normalized to the muscle area from muscle 6/7 NMJ at A2 hemisegment in control animals (1.33 ± 0.03), AP2σKG02457/AP2σang7 (2.50 ± 0.11), and witA12, AP2σKG02457/AP2σang7 (2.0 ± 0.07). Error bar represents SEM; the statistical analysis was done using one-way ANOVA followed by post hoc Tukey’s test. **p < 0.01, ***p < 0.001. Download Figure 1-1, TIF file. [file enu-eN-NWR-0044-22-s01.tif]

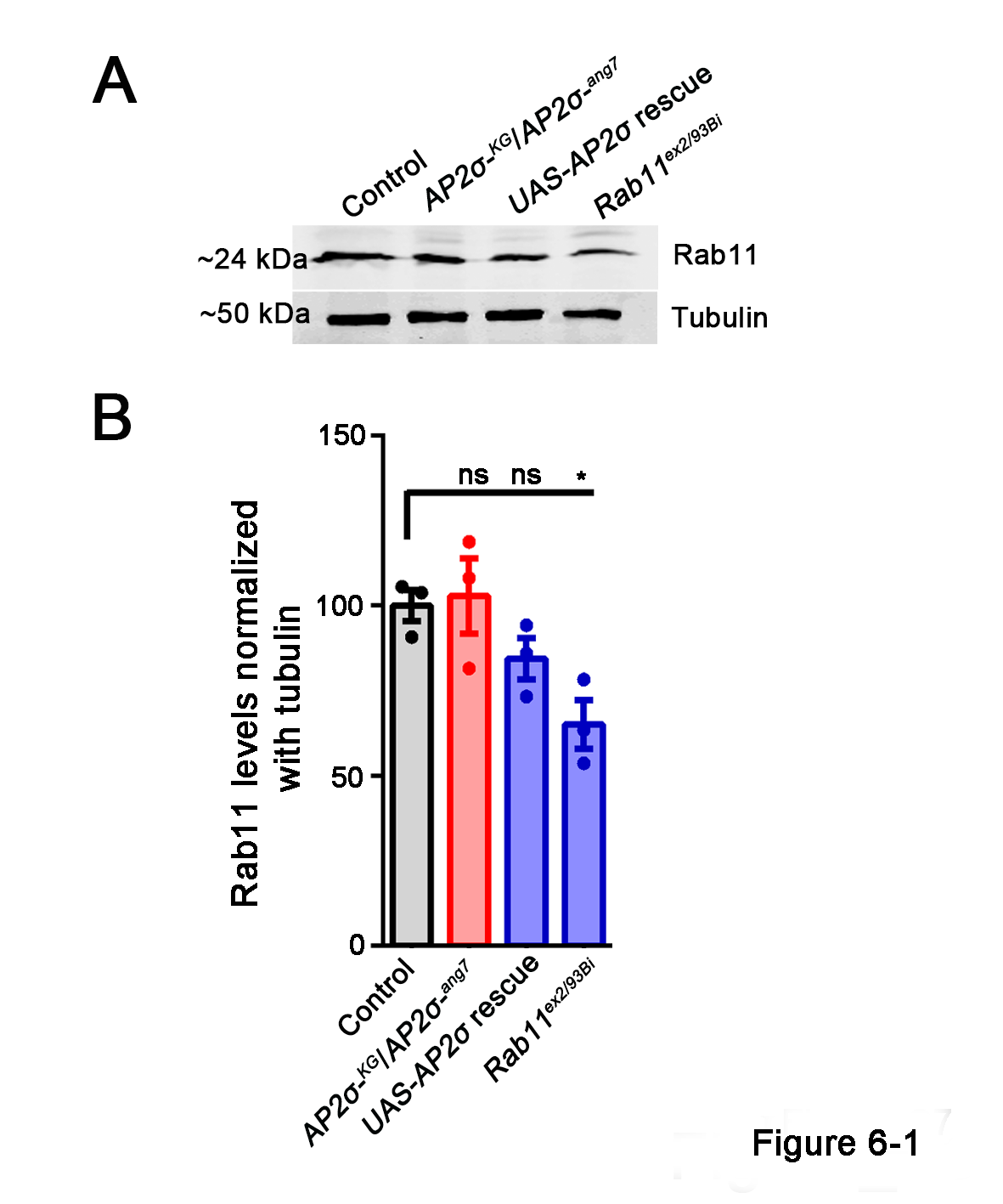

Supplement: Extended Data Figure 6-1 — σ2 adaptin mutants have normal Rab11 protein levels. A, Western blotting shows the levels of total Rab11 for control, AP2σKG02457/AP2σang7, D42-Gal4, AP2σang7/UAS- AP2σ, AP2σKG02457, and Rab11ex2/93Bi. Note that total Rab11 protein levels are identical across all genotypes except Rab11ex2/93Bi. B, Histogram showing the average levels of Rab11 normalized with internal control tubulin for control (100 ± 4.65), AP2σKG02457/AP2σang7 (102.8 ± 11.07), D42-Gal4, AP2σang7/UAS- AP2σ, AP2σKG02457 (84.45 ± 6.11), and Rab11ex2/93Bi (65.06 ± 7.16). Error bar represents SEM; the statistical analysis was done using one-way ANOVA followed by post hoc Tukey’s test. *p < 0.05; ns, not significant. Download Figure 6-1, TIF file. [file enu-eN-NWR-0044-22-s02.tif]

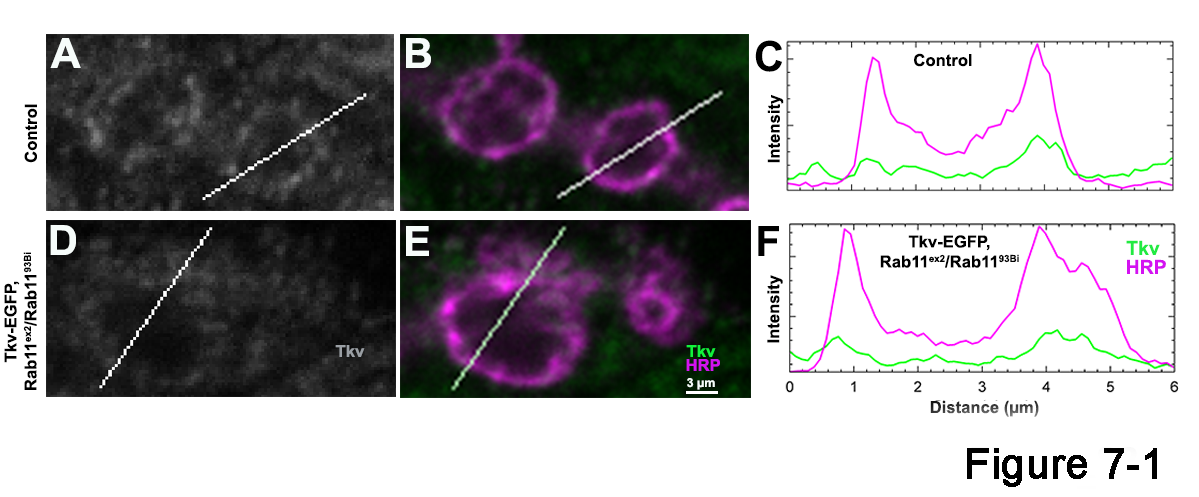

Supplement: Extended Data Figure 7-1 — Tkv-EGFP is not localized at the presynaptic membrane in the Rab11 mutants. A–F, A single confocal section of a bouton labelled for Tkv (represented in grayscale) and presynaptic membrane marker HRP (magenta) in D42-Gal4/UAS-tkv-EGFP (A, B) and D42-Gal4/UAS-tkv-EGFP (D, E). The intensity profile across the bouton in D42-Gal4/UAS-tkv-EGFP (F) suggests that Tkv is not enriched at the presynaptic membrane. Download Figure 7-1, TIF file. [file enu-eN-NWR-0044-22-s03.tif]

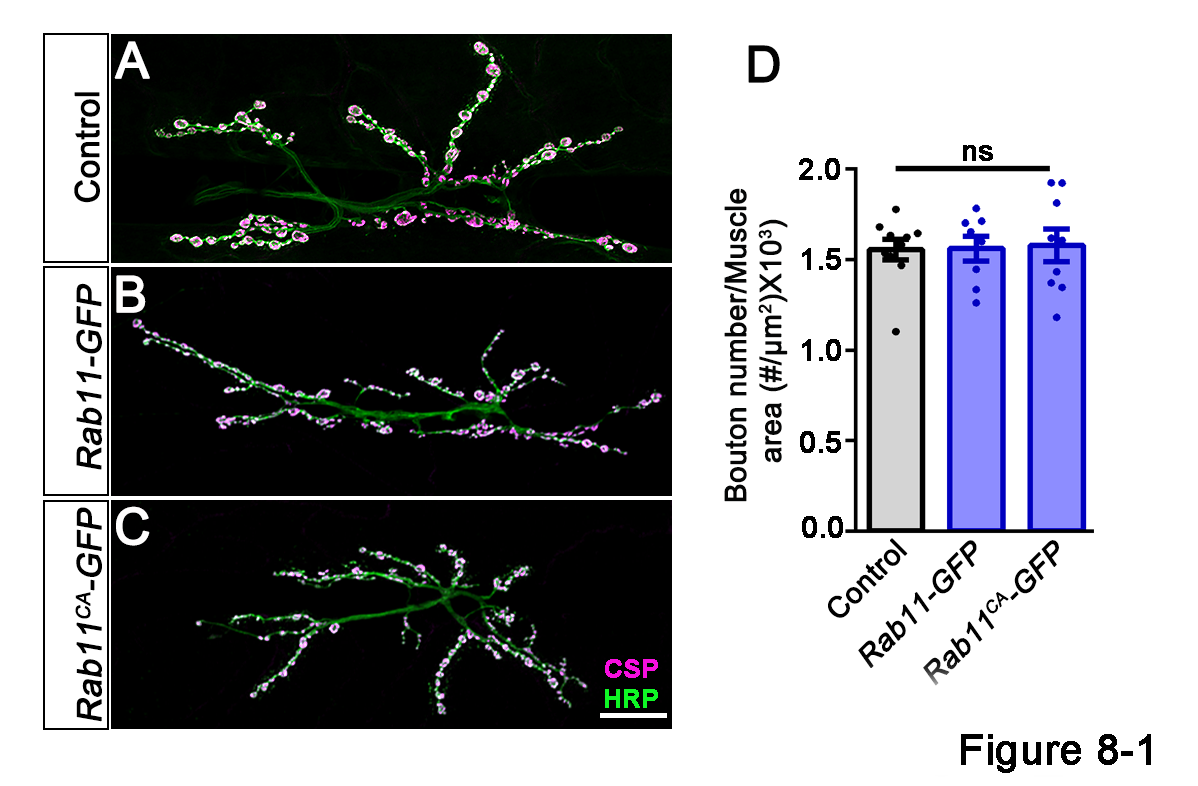

Supplement: Extended Data Figure 8-1 — Expressing a wild-type or constitutively active form of Rab11 does not alter the synaptic morphology. A–C, Confocal images of NMJ synapses at muscles 6/7 NMJ at A2 hemisegment showing the synaptic growth in control (A), D42-Gal4-driven UAS-Rab11-GFP (B), and D42-Gal4-driven UAS-Rab11Q70L-GFP (C) double immunolabeled with a presynaptic vesicle marker, CSP (magenta), and a neuronal membrane marker, HRP (green), to mark the bouton outline. Expressing the wild-type and active form of Rab11 does not show morphological changes at the synapse. Scale bar in C represents 20 μm. D, Histogram showing the average bouton number normalized to the muscle area from muscle 6/7 NMJ at A2 hemisegment in control animals (1.56 ± 0.06), UAS-Rab11-GFP/+; D42-Gal4/+ (1.56 ± 0.07), and UAS-Rab11Q70L-GFP; D42-Gal4/+ (1.58 ± 0.09). Error bar represents SEM; the statistical analysis was done using one-way ANOVA followed by post hoc Tukey’s test. ns, not significant. Download Figure 8-1, TIF file. [file enu-eN-NWR-0044-22-s04.tif]

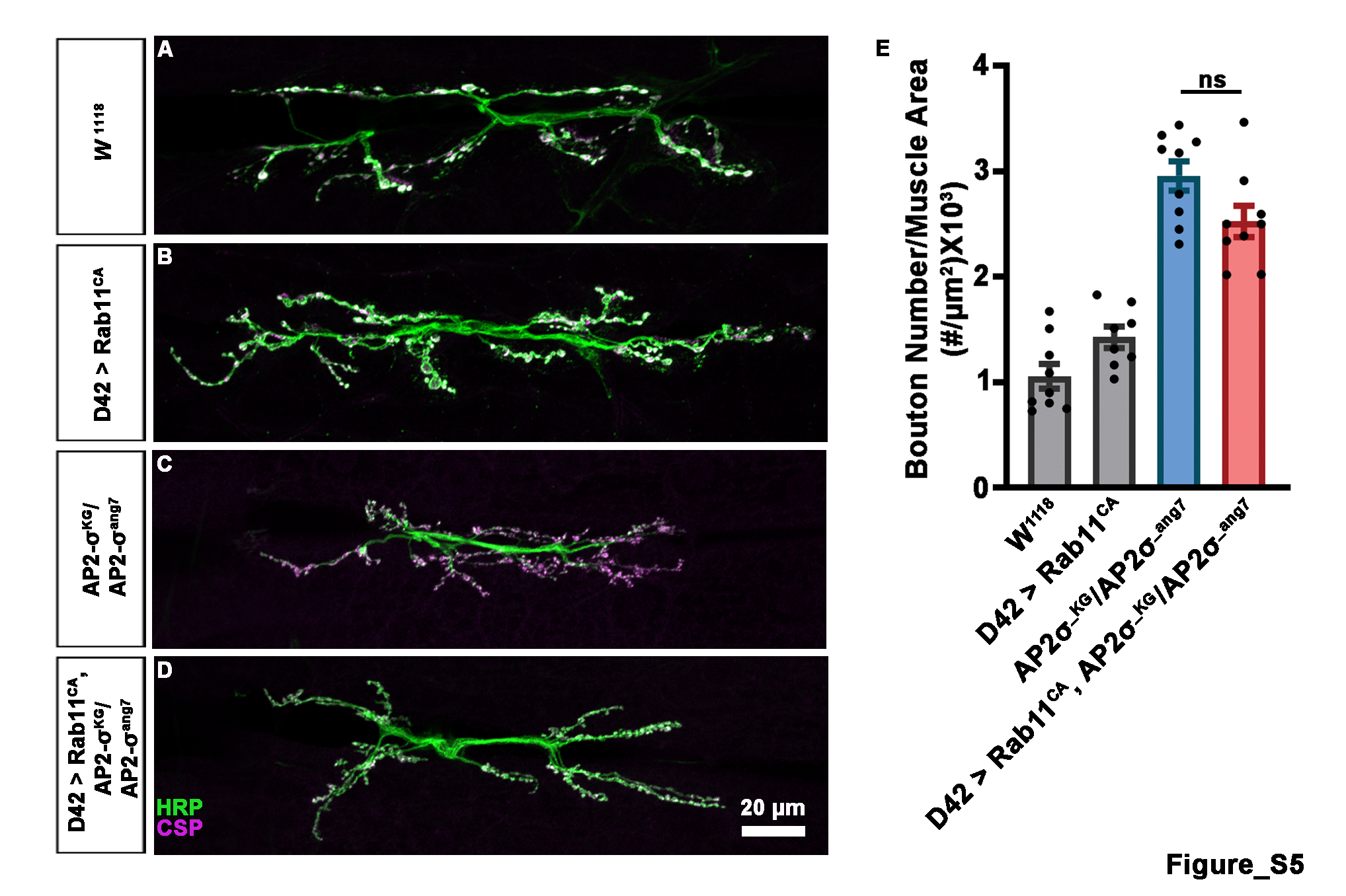

Supplement: Extended Data Figure 8-2 — Constitutively active Rab11 does not restore the NMJ morphological defects of σ2-adaptin mutants. A–D, Confocal images of NMJ synapses at muscle 6/7 NMJ at A2 hemisegment showing synaptic growth in (A) Control animals, (B) D42-Gal4 > Rab11CA, (C) AP2σKG02457/AP2σang7, and (D) D42-Gal4 > Rab11CA, AP2σang7/AP2σKG02457 double immunolabeled with a presynaptic synaptic vesicle marker, CSP (magenta) and a neuronal membrane marker, HRP (green) to mark the bouton outline. Scale bar in D represents 20 μm. E, Histogram showing average bouton number normalized to the muscle area from muscle 6/7 NMJ at A2 hemisegment in control animals (1.05 ± 0.11), D42-Gal4 > Rab11CA (1.4 ± 0.10), AP2σKG02457/AP2σang7 (2.9 ± 0.14), and D42-Gal4 > Rab11CA, AP2σang7/AP2σKG02457 (2.5 ± 0.14). Error bar represents SEM; statistical analysis was done using one-way ANOVA followed by post hoc Tukey’s test. **p < 0.01; ns, not significant. Download Figure 8-2, TIF file. [file enu-eN-NWR-0044-22-s05.tif]

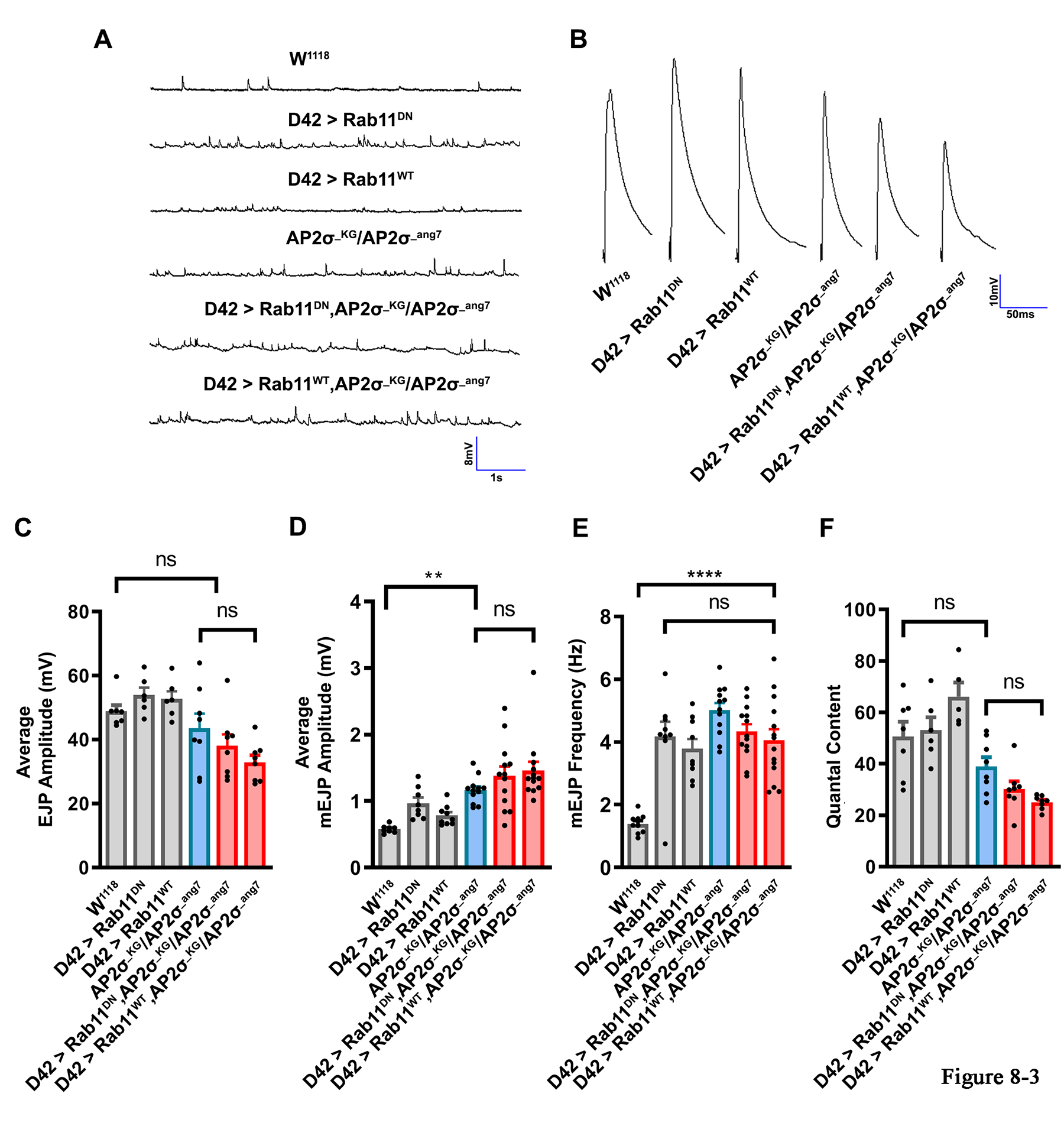

Supplement: Extended Data Figure 8-3 — Overexpression of Rab11 does not restore the functional defects of σ2-adaptin. A, Representative traces of mEJP in control, D42 > Rab11DN, D42 > Rab11WT, heteroallelic AP2σKG02457/AP2σang7, D42 > Rab11DN, AP2σKG02457/AP2σang7, and D42 > Rab11WT, AP2σKG02457/AP2σang7 larvae. B, Representative tracesof EJP in control, D42 > Rab11DN, D42 > Rab11WT, heteroallelic AP2σKG02457/AP2σang7, D42 > Rab11DN, AP2σKG02457/AP2σang7, and D42 > Rab11WT, AP2σKG02457/AP2σang7 larvae. C, Quantification of average EJP amplitude in control (44.42 ± 1.93), D42 > Rab11DN (53.89 ± 2.35), D42 > Rab11WT (52.71 ± 2.42), heteroallelic AP2σKG02457/AP2σang7 (43.57 ± 4.52), D42 > Rab11DN, AP2σKG02457/AP2σang7 (38.03 ± 3.60), and D42 > Rab11WT, AP2σKG02457/AP2σang7 (32.92 ± 2.14). Error bars represent SEM; statistical analysis is based on one-way ANOVA followed by post hoc Tukey’s multiple-comparison test. **p < 0.01; ns, not significant. D, Quantification of average mEJP amplitude in control (0.56 ± 0.01), D42 > Rab11DN (0.96 ± 0.08), D42 > Rab11WT (0.78 ± 0.05), heteroallelic AP2σKG02457/AP2σang7 (1.19 ± 0.05), D42 > Rab11DN, AP2σKG02457/AP2σang7 (0.13 ± 0.14), and D42 > Rab11WT, AP2σKG02457/AP2σang7 (1.46 ± 0.13). Error bars represent SEM; statistical analysis is based on one-way ANOVA followed by post hoc Tukey’s multiple-comparison test. **p < 0.01; ns, not significant. E, Histograms showing average mEJP frequency in control (1.38 ± 0.10), D42 > Rab11DN (4.17 ± 0.47), D42 > Rab11WT (3.78 ± 0.31), heteroallelic AP2σKG02457/AP2σang7 (5.01 ± 0.23), D42 > Rab11DN, AP2σKG02457/AP2σang7 (4.33 ± 0.23), and D42 > Rab11WT, AP2σKG02457/AP2σang7 (4.06 ± 0.34). Error bars represent SEM; statistical analysis is based on one-way ANOVA followed by post hoc Tukey’s multiple-comparison test. **p < 0.01; ns, not significant. F, Quantification of quantal content in control (50.56 ± 5.82), D42 > Rab11DN (53.14 ± 4.91), D42 > Rab11WT (66.11 ± 5.48), heteroallelic AP2σKG02457/AP2σang7 (38.95 ± 3.65), D42 > Rab11DN, [file enu-eN-NWR-0044-22-s06.tif]

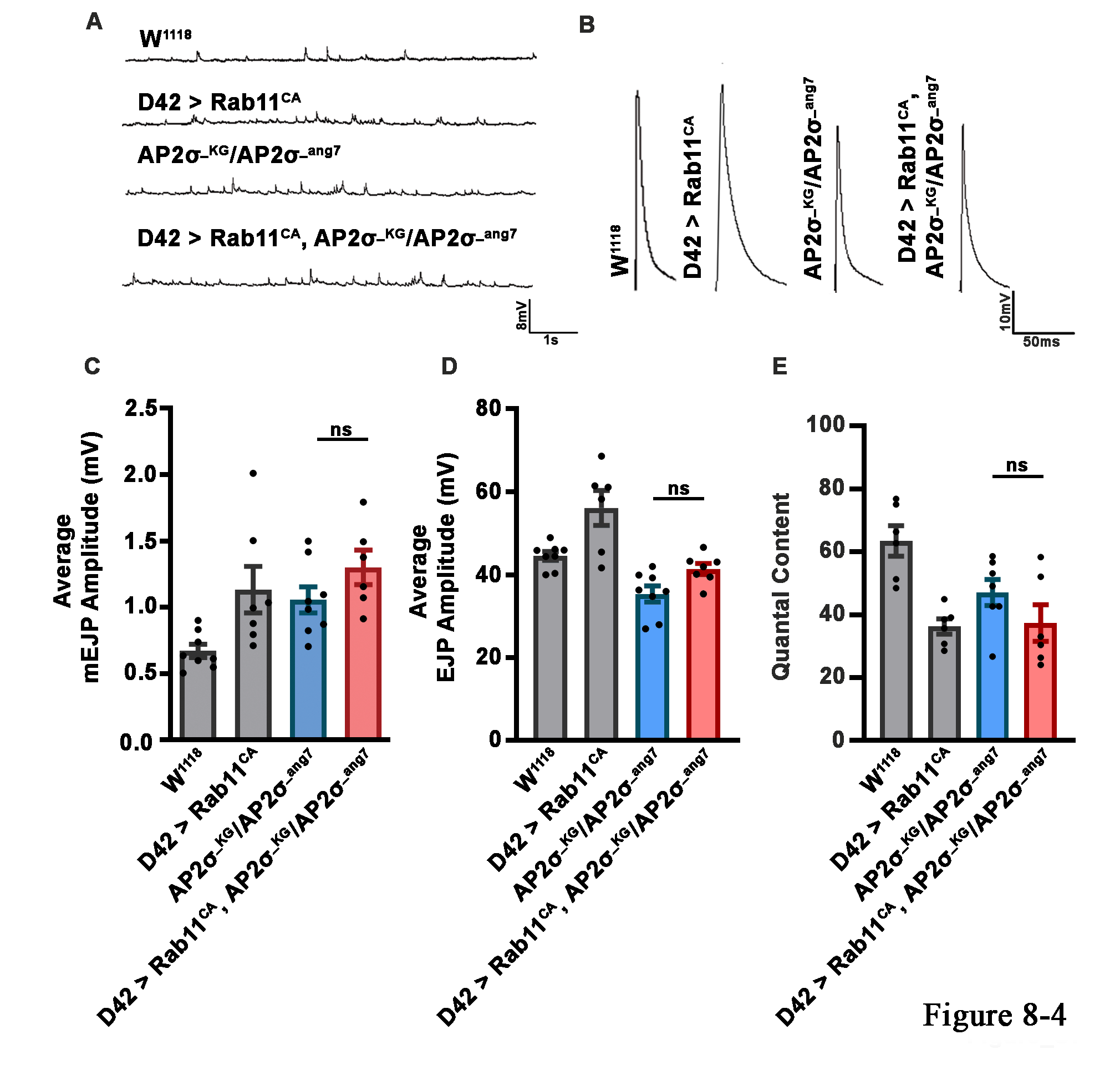

Supplement: Extended Data Figure 8-4 — Constitutively active Rab11 does not restore the physiological defects of σ2-adaptin mutants. A, Representative traces of mEJP in control, D42-Gal4 > Rab11CA, heteroallelic AP2σKG02457/AP2σang7, and D42-Gal4 > Rab11CA, AP2σang7/AP2σKG02457 larvae. B, Representative traces of EJP in control (w1118), D42-Gal4 > Rab11CA, heteroallelic AP2σKG02457/AP2σang7, and D42-Gal4 > Rab11CA, AP2σang7/AP2σKG02457 larvae. C, Quantification of average mEJP amplitude (mV) in control (0.67 ± 0.04), D42-Gal4 > Rab11CA (1.13 ± 0.17), heteroallelic AP2σKG02457/AP2σang7 (1.05 ± 0.09) and D42-Gal4 > Rab11CA, AP2σang7/AP2σKG02457 (1.30 ± 0.13). Error bars represent SEM; statistical analysis is based on one-way ANOVA followed by post hoc Tukey’s multiple-comparison test. **p < 0.01; ns, not significant. D, Quantification of average EJP amplitude in control (44.52 ± 1.07), D42-Gal4 > Rab11CA (56.85 ± 4.221), heteroallelic AP2σKG02457/AP2σang7 (35.40 ± 1.925) and D42-Gal4 > Rab11CA, AP2σang7/AP2σKG02457 (41.41 ± 1.340). The error bars represent the SEM; statistical analysis is based on one-way ANOVA followed by post hoc Tukey’s multiple-comparison test. **p < 0.01; ns, not significant. E, Histograms showing quantal content in the control (63.43 ± 4.81), D42-Gal4 > Rab11CA (26.88 ± 4.42), heteroallelic AP2σKG02457/AP2σang7 (47.08 ± 4.13) and D42-Gal4 > Rab11CA, AP2σang7/AP2σKG02457 (37.38 ± 5.83). The error bars represent the SEM; statistical analysis is based on one-way ANOVA followed by post hoc Tukey’s multiple comparison test. **p < 0.01; ns, not significant. Download Figure 8-4, TIF file. [file enu-eN-NWR-0044-22-s07.tif]
